# Integrating artificial intelligence and manual curation to enhance bioassay annotations in ChEMBL

**DOI:** 10.1186/s13321-026-01165-x

**Published:** 2026-02-12

**Authors:** Ines Smit, Melissa F. Adasme, Emma Manners, Sybilla Corbett, Nicolas Bosc, Hoang-My-Anh Do, Andrew R. Leach, Noel M. O’Boyle, Barbara Zdrazil

**Affiliations:** 1 European Molecular Biology Laboratory, European Bioinformatics Institute (EMBL-EBI), Wellcome Genome Campus, Hinxton, Cambridgeshire, CB101SD UK; 2 Present Address: LifeArc, Accelerator Building, Open Innovation Campus, Stevenage, SG1 2FX UK

**Keywords:** ChEMBL, FAIR data, Bioassay annotation, Natural language processing, Named entity recognition, Machine learning, Experimental method, Bioassay Ontology

## Abstract

As the volume and diversity of bioactivity data in ChEMBL continues to grow, ensuring that assay metadata is standardized, interoperable, and machine-readable is critical for effective use in cheminformatics and ML applications. In this work, we present recent efforts to enhance the quality and granularity of bioassay annotations in ChEMBL through a combination of manual and semi-manual curation and AI-driven approaches. We introduce a “perfect assay description” template to guide consistent annotation and demonstrate how natural language processing techniques and multi-class classification can be used to automatically extract key assay parameters and assign broad assay categories for legacy data. We report on the development, validation, and application of a spaCy-based NER model that identifies experimental methods with high precision and recall, as well as a complementary classification model that refines ASSAY_TYPE categorization beyond the existing schema. In addition, we describe improvements to metadata extraction for ADME endpoints, organism and protein variant annotations, and ontology linking using tools such as text2term. Together, these enhancements significantly advance the FAIRness of ChEMBL’s bioassay data, enabling more robust downstream analyses and more precise compound-target activity modeling.

**Graphical Abstract:**

**Figure:**
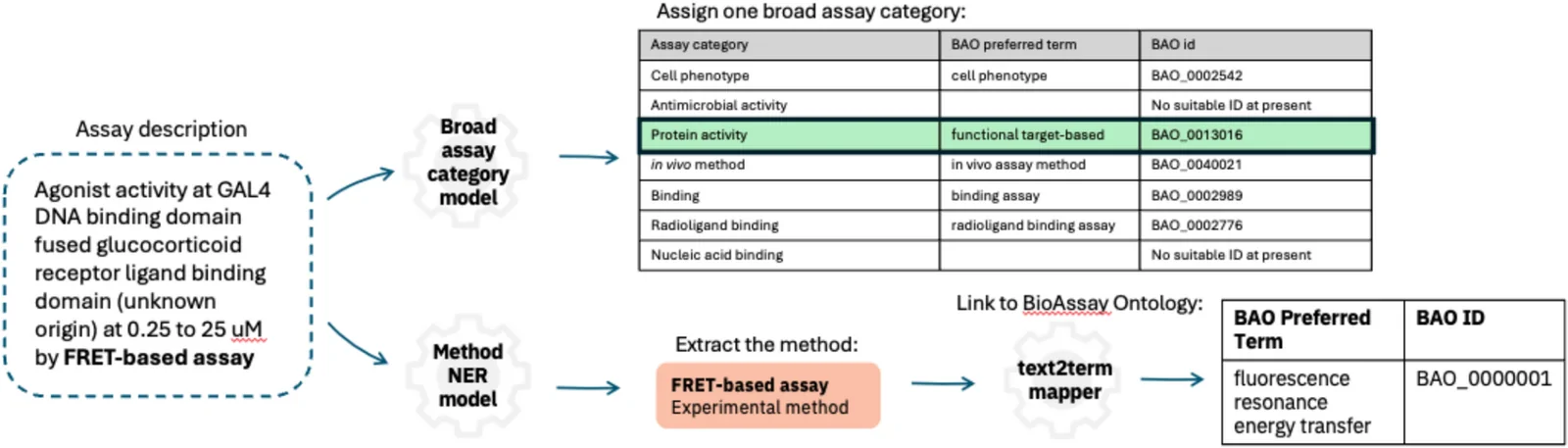

**Supplementary Information:**

The online version contains supplementary material available at https://doi.org/10.1186/s13321-026-01165-x.

## Scientific Contribution

We present manual and AI-based methods to improve the annotation and classification of bioassays in ChEMBL, including large-scale extraction of experimental methods and refined assay categorization. These enhancements increase the interpretability and reusability of bioactivity data, supporting more informed dataset selection for predictive modeling. The resulting tools and annotations have been made openly available and will directly benefit the cheminformatics and drug discovery communities by enabling more consistent and FAIR-compliant use of ChEMBL data.

**Supplementary Information:**

The online version contains supplementary material available at https://doi.org/10.1186/s13321-026-01165-x.

## Introduction

With an ever-growing volume of bioassay data stored in open access databases such as ChEMBL [1] and an increasing interest in using bioactivity data in large-scale analyses and machine learning (ML) applications, the interoperability and reusability aspects of the FAIR (findable, accessible, interoperable, and reusable) principles [2] are gaining particular attention. Assay parameters need to be properly captured in data repositories, which means that certain minimum information required to interpret an assay setup needs to be available (accessible). In addition, the information needs to be presented in a structured format with stable identifiers (findable), ideally linked to rich metadata aligned with scientific terminology via domain-specific ontologies. The latter satisfies the interoperability and reusability requirements.

Several initiatives have the goal of establishing guidelines for describing bioactive entities and for harmonising bioassay data. Importantly, the MIABE (Minimum Information About a Bioactive Entity) checklist for reporting bioactive molecules outlines essential metadata—chemical identity, biological assays, ADMET (Absorption, Distribution, Metabolism, Excretion, and Toxicity) properties, provenance, etc.—to promote reproducibility, data integration, and mining across drug discovery research [3].

BARD (BioAssay Research Database) was a semantic, curated repository of high-throughput screening (HTS) assay metadata based on FAIR principles, established with U.S. NIH funding to improve the annotation, integration, and reusability of biological assay data. One of the projects’ aims was to enrich PubChem BioAssay data [4] with better metadata. Unfortunately, BARD as a standalone platform is no longer active (as of the mid-2010s) but its concepts live on in some of PubChem’s data and in the current structure of the Bioassay Ontology (BAO) [5, 6]. Indeed, BARD adopted BAO as its core semantic framework, using its controlled vocabularies. The BARD project contributed feedback and refinements to BAO based on their real-world assay curation needs, which in some cases led to an expansion of BAO [7].

Bioassay annotation was also investigated in the framework of the public private partnership formed as part of the Open PHACTS consortium [8], particularly with respect to using nanopublications to describe how comparable are two biassays stored in ChEMBL.

Further efforts have demonstrated the feasibility of producing structured assay metadata at scale. The Data FAIRy project from the Pistoia Alliance illustrates the value of combining machine learning with expert curation for drug discovery: through NLP processing of assay protocols, ontology-based standardization, and expert review, the project shows that structured assay metadata can be semi-automatically extracted and selected annotations have been deposited to PubChem [9].

In parallel, the Drug Target Commons (DTC) initiative [10] introduced a community-driven platform to harmonise drug–target bioactivity data. A central innovation of DTC was the use of “micro-annotations” derived from BAO to capture fine-grained assay context—such as assay format, detection technology, endpoint type, and biological system—which enabled more consistent comparison and integration of heterogeneous bioactivity measurements. While broader community curation in DTC appears less active today, the micro-annotation concept has been influential in demonstrating the value of detailed ontology-backed assay context for FAIRification and benchmarking of drug–target data.

Complementary to these efforts, the MICHA (Minimal Information for Chemosensitivity Assays) project [11] proposed a minimal information standard and software pipeline for curating and FAIRifying chemosensitivity datasets, combining structured ontologies, protocol capture, and automated extraction of compound and sample metadata. MICHA highlighted the practical need for standardized assay metadata as drug screening datasets increase in scale and importance.

Developments in assay annotation in the commercial sector (e.g., platforms built by pharmaceutical companies or cheminformatics vendors) are more difficult to follow and adopt for the academic and open-data communities. Eventually, parts of these commercial technologies may be released to the public, as in the case of BioAssay Express [12, 13], developed by Collaborative Drug Discovery (CDD).

Assay metadata, especially in free-text form holds valuable contextual information. Recently, research published by Schoenmaker et al. showed that embeddings derived from natural language processing (NLP) of ChEMBL assay descriptions can effectively encode this context and thus improve the performance and interpretability of bioactivity prediction models in certain datasets [14]. The authors recommend that future bioactivity datasets should standardize and enrich assay metadata to enable such modeling strategies.

The ChEMBL team has been working towards the goals of FAIR data sharing since its inception in 2009. The main elements of ChEMBL’s biological data curation pipeline are described in Papadatos et al. [15] and are therefore not the focus of this article. Herein, the current state of the art of ChEMBL’s assay structure and assay data representation are presented. We summarise available data annotations for assays, which are crucial for a detailed understanding of compound-target bioactivity measurements. We highlight more recent efforts in the ChEMBL team such as the use of modern techniques of NLP to extract additional annotations from assay descriptions such as the experimental method used in an assay protocol.

Bioactivity data analyses can suffer when bioactivity data points from disparate assays are combined [16, 17]. A key advantage of an improved assay annotation to the Cheminformatics and Computational Chemistry communities will be to enable users of ChEMBL data to make better informed decisions about different categories of bioassay data within the database without the need to consult the primary literature. It will nevertheless remain the user’s responsibility to decide which criteria to apply when combining bioactivity data from distinct assays, for example when building ML models. However, as available annotations for bioassays become richer and more organised, rules for selecting bioassay data can be more easily implemented and automated.

In the following sections, the current representation of bioassay data in ChEMBL is outlined, with particular attention to the tables and fields used for its storage, as well as the established data annotations.

### Structured bioassay data in ChEMBL

**Fig. 1:**
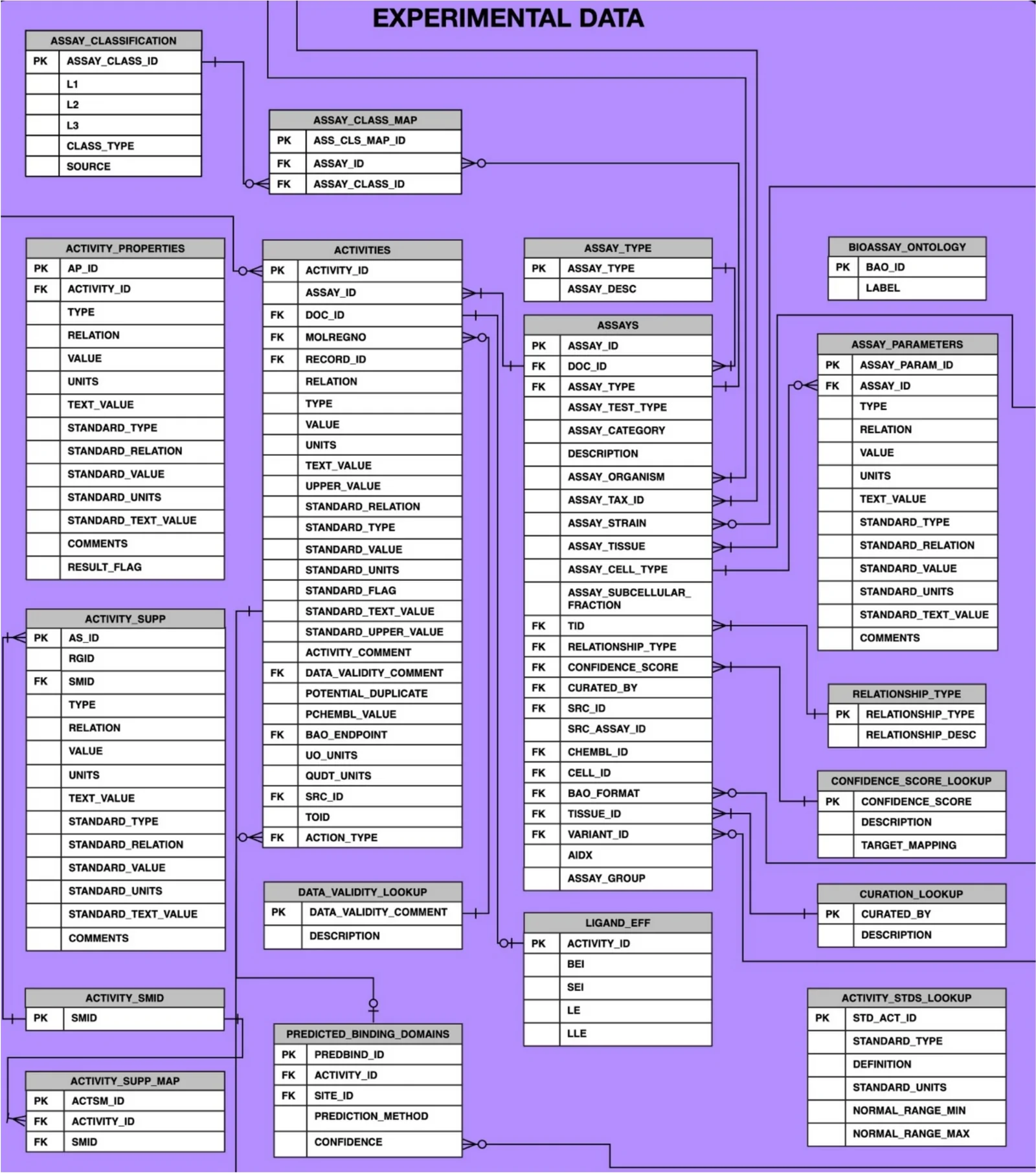
Entity-relationship diagram (ERD) showing the ChEMBL 35 tables storing experimental data (extracted from the ChEMBL FTP site [27])

ChEMBL stores information about bioassays and corresponding bioactivity measurements in the ASSAYS and ACTIVITIES tables (Fig. 1), respectively, together with relevant parameters that enable assays to be compared. The main high-level entities describing an assay are information about the compound tested, target, and assay conditions; alongside the corresponding bioactivity measurements. It is important to note that the definition of a “target” in ChEMBL covers both molecular and non-molecular targets. Molecular targets are primarily proteins with some nucleic acids and small molecules; non-molecular targets refer to assays with cell lines, tissues, or whole organisms as the main target.

Over the years, the ChEMBL team has created additional value by extracting and curating more fine-tuned (meta-)data for bioassays. Examples include data on protein variation, assay strain, organism taxonomy ID, cell type, subcellular fraction, and certain measures to evaluate the confidence of assay-target mapping. These data are semi-manually curated, mapped to suitable ontologies (such as UniProt for proteins [18]; Cell Line Ontology [19], Experimental Factor Ontology (EFO) [20], Cellosaurus [21], Cell Ontology [22], and LINCS cell dictionary for cells [23]; and Uberon [24], EFO, Brenda Tissue Ontology [25] and CALOHA for tissues [26]), and stored in specific dictionaries that map to the ASSAYS table. However, some data types can also be found in dedicated metadata tables, such as the ASSAY_PARAMETERS, ACTIVITY_PROPERTIES, and ACTIVITY_SUPP tables (introduced with ChEMBL releases 18, 24, and 24, respectively). These metadata tables provide additional context for ASSAYS and their associated ACTIVITIES with the aim of providing more meaningful data in ChEMBL. The ASSAY_PARAMETERS table includes metadata such as the dose, administration route, and time point of measurement. Metadata in the ASSAY_PARAMETERS table is annotated at the ASSAY level and therefore applies to all ACTIVITIES associated with an ASSAY. Similar information is captured in the ACTIVITY_PROPERTIES table (i.e., dose or time points, or additional data related to an activity readout such as a Hill slope) but, in this case, allows annotation of individual activity data points (mapped to the same assays) with differing metadata. Together, these tables offer depositors some flexibility; there is a choice whether to record a single assay alongside multiple activities that differ in their metadata (captured in the ACTIVITY_PROPERTIES table) or to submit a larger number of assays with assay-level metadata specified in the ASSAY_PARAMETERS table resulting in fewer activities mapped to each distinct assay.

Publicly available tables storing information related to the experimental data are shown in Fig. 1. More in-depth information about the respective target and compound measured in an assay can be found in separate tables [27].

### Assay descriptions

In addition to providing bioassay parameters in bespoke database fields and annotation with a structured vocabulary, a free-text description of the assay conditions is also provided (stored in the ASSAYS table as DESCRIPTION). Even though the ChEMBL team has recently provided guidance on how to construct assay descriptions for data depositors [28], this field was originally created with flexibility in mind. It is challenging to follow a very strict format given the huge diversity of assays that have been stored in ChEMBL over time [1]. Conversely, analysing how descriptions of assays vary and how well they capture what has been described in the original publication for data extracted from literature is a timely undertaking, especially given the current interest in using NLP techniques to capture assay parameters from the literature text directly. Thus, the first step towards better assay annotations consisted of defining a “perfect assay description” for data extracted from scientific literature in ChEMBL. This helped us improve our public documentation and give feedback to our data extractors. These efforts will also help to make future assay descriptions more consistent and well-structured which in turn will facilitate automated (NLP-based) entity extraction going forward.

### ASSAY_TYPE categories in ChEMBL

**Fig. 2:**
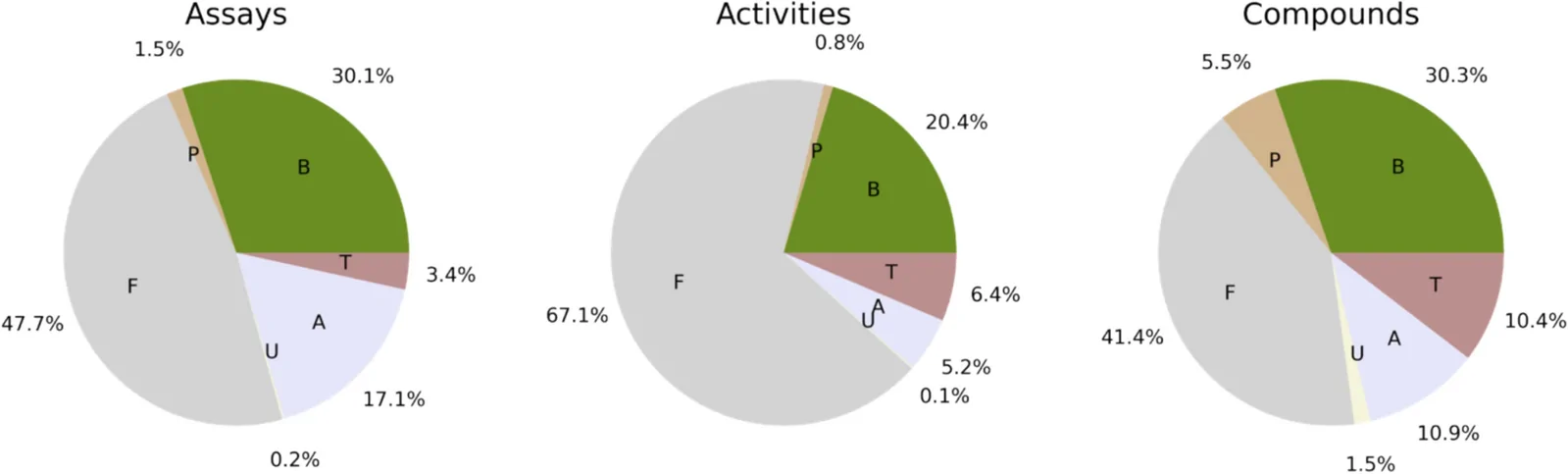
Share of different ASSAY_TYPE categories in ChEMBL 35. The percentage of assays, activities, and compounds belonging to each category are shown

One of the broadest categorisations of assays in ChEMBL, is the ASSAY_TYPE (stored in the ASSAYS table) which is assigned during the processes of data extraction from literature and reviewed during subsequent data curation. Currently, ASSAY_TYPE can take one of six categories (resembling the bioassay types in the Bioassay Ontology): “B” (“Binding”)—data measuring binding of a compound to a molecular target, e.g. K_i_, IC_50_, Kd; “F” (“Functional”)—data measuring the biological effect of a compound, e.g. %cell death in a cell line, rat weight; “A” (“ADME”)—ADME data e.g. t_1/2_, oral bioavailability; “T” (“Toxicity”)—data measuring toxicity of a compound, e.g., cytotoxicity; “P” (“Physicochemical”)—assays measuring physicochemical properties of the compound(s) in the absence of biological material e.g., chemical stability, solubility; and “U” (“Unclassified”)—a small proportion of assays which cannot be classified into one of the other categories e.g., ratio of binding vs efficacy. Historically, ASSAY_TYPE categories B, F, and A were introduced first (with the very first release, ChEMBL 01). With ChEMBL 13, the first P-type assays entered ChEMBL, and from ChEMBL 18 onwards toxicity assays were classified as type T, rather than being part of the A-category. Figure 2 shows the share of ASSAY_TYPE categories in ChEMBL 35, with a clear predominance of functional assays (47.7% of all assays, 67.1% of all activities, and 41.4% of all compounds being assigned to category F). Importantly, while the ASSAY_TYPE categories are not mutually exclusive, each assay is assigned to only one category. To give an example, an assay described as “Inhibition of human Cytochrome P450 3A4” could in principle be classified as both a F-type as well as an A-type assay. However, we typically categorise those as ADME assays, since we believe this is the more useful categorisation for users.

### Classification of in vivo assays in ChEMBL

From ChEMBL 24 onwards, in vivo assays have been systematically identified and annotated with their animal disease model or phenotypic output for every ChEMBL release as described in Hunter et al. [29]. This information is stored in the ASSAY_CLASSIFICATION table. These data currently comprises ~ 126,000 annotated in vivo assays for ChEMBL 35. The reference animal models or disease/phenotypic endpoints are routinely mapped to Medical Subject Heading (MeSH) ontology terms, which enables researchers to identify compounds that have been investigated for a group of disease-, pharmacology- or toxicity-relevant assays.

### BAO endpoint and BAO assay format annotations

Previous work towards aligning ChEMBL assays and activities to an ontology used the Bioassay Ontology (BAO) [5]; ChEMBL standard activity types were mapped to the corresponding BAO_ENDPOINT terms (stored in the ACTIVITIES table as BAO_ENDPOINT) and assays were mapped to BAO_FORMAT terms (stored in the ASSAYS table as BAO_FORMAT).

Mapping to BAO_ENDPOINT terms was achieved for 79% of all bioactivities reported in ChEMBL 35. The remaining 21% of activities are mapped to the term “result” (BAO_0000179) as a placeholder for any endpoints that do not map to our current (manually created) dictionary. Table 1 only shows the fifteen most prevalent BAO_ENDPOINT categories.

**Table 1:** Assay/activity counts for different BAO_FORMAT categories and BAO_ENDPOINT categories based on ChEMBL 35 (the table shows the 15 top categories for BAO_ENDPOINT)

BAO_FORMAT	LABEL	Assays	BAO_ENDPOINT	LABEL	Activities
BAO_0000218	Organism-based format	630,320	BAO_0000186	AC50	4,773,689
BAO_0000219	Cell-based format	558,405	BAO_0000179	result	4,435,183
BAO_0000357	Single protein format	242,687	BAO_0000190	IC50	2,802,898
BAO_0000019	Assay format	181,989	BAO_0000189	GI50	2,623,650
BAO_0000221	Tissue-based format	28,066	BAO_0000201	Percent inhibition	1,529,105
BAO_0000100	Small-molecule physicochemical format	25,587	BAO_0000192	Ki	775,444
BAO_0000251	Microsome format	18,896	BAO_0002146	MIC	748,009
BAO_0000366	Cell-free format	14,414	BAO_0001103	Percent activity	648,861
BAO_0000223	Protein complex format	92,77	BAO_0000375	Fold activation	625,438
BAO_0000249	Cell membrane format	8546	BAO_0000188	EC50	522,752
BAO_0000224	Protein format	8491	BAO_0000034	Kd	186,024
BAO_0000220	Subcellular format	6566	BAO_0000181	Response endpoint	134,463
BAO_0000225	Nucleic acid format	5902	BAO_0000193	Fold change	115,800
BAO_0000252	Mitochondrion format	1281	BAO_0003036	ED50	103,415
BAO_0000217	Biochemical format	119	BAO_0003039	IZ	103,083

Supplementary Table S1 contains descriptions for the BAO_FORMAT and BAO_ENDPOINT terms used

The BAO_FORMAT annotations were generated by a rule-based approach based on information in the assay DESCRIPTION as well as taking the assigned target into account. These annotations allow users to distinguish fifteen categories, with “organism-based format” and “cell-based format” being the most prevalent categories to date (Table 1). This potentially points to an increasing number of assays in ChEMBL with non-protein based targets [1], or alternatively to an increasing number of assays testing protein activity in cell-based assays or in vivo/ex vivo models. For BAO_FORMAT mappings, 10.7% of all assays currently cannot be mapped to a more specific term than “assay format” (BAO_0000019).

While there is certainly space for updating some of the existing BAO annotations in order to capture a larger percentage of activities and assays, the current focus for improving the assay annotations in ChEMBL is to enhance the BAO annotations with additional relevant entities (i.e. falling under additional categories, other than the BAO_FORMAT or BAO_ENDPOINT).

## Results

Here, we describe most recent enhancements to annotate bioassay parameters in ChEMBL, as described in the introduction, by using a combination of manual expert curation and artificial intelligence (AI) approaches. We introduce the concept of a “perfect assay description”, describe the enhanced curation of assays for recent releases of ChEMBL, and we also show examples of the use of text mining and AI approaches to extract additional relevant assay parameters from the assay DESCRIPTION.

### Towards a “perfect assay description”

Bioactivity data types in ChEMBL have diversified over time [1], but in general the two main classes of documents containing the information are of type “PUBLICATION” (extracted from the scientific literature) and “DATASET” (provided directly by depositors). Whereas we continue to work closely with depositors to ensure the submitted assay information meets our minimum criteria and follows our general structure for data deposition, for literature sources the submitted data is more uniform in general. This allows us to use a high-level template for constructing the assay DESCRIPTION for literature data which consists of the following components:

$$\begin{gathered} Activity \, of \, drug \, + \, Target/Biological \, system \, + \, Expression \, system \, + \, Inducing \, agent \, + \, Assessment \, \hfill \\ + \, Dose \, + \, Dose \, unit \, + \, Route \, + \, Drug \, Regime \, + \, Incubation \, time \, + \, Method \, + \, Miscellaneous \, data \hfill \\ \end{gathered}$$

However, the “perfect assay description” will vary depending on the nature of the assay (i.e., ASSAY_TYPE). For example, a precise record of a purified protein target (source organism, amino acid changes, deletions) is critical for binding assays whereas the route of drug administration is more relevant to pharmacokinetics studies (ADME assays). Furthermore, the parameters necessary for interpretation vary according to the specific assay; a record of the solvent, pH and temperature (but not necessarily a target) is essential for drug stability assays whereas information on drug metabolites is important for metabolism assays and the target tissue (as well as biological system) may be key to understanding toxicity assays. For certain functional and binding assays, specific parameters, such as the voltage protocol for electrophysiology assays with ion channel targets such as hERG, improves interpretation [30].

### Storing assay details for deposited data sets

While for literature data relatively well-structured assay descriptions are usually submitted to ChEMBL from data extraction process, direct data depositions—in general—provide assay descriptions with a more flexible structure according to project requirements. Similarly, there is also flexibility in the parameters included within the assay DESCRIPTION and in the metadata tables (ASSAY_PARAMETERS and ACTIVITY_PROPERTIES). However, deposited data now makes up 53% of bioactivity data within ChEMBL (9.99 M bioactivity data points for “PUBLICATIONS” vs 11.41 M data points for “DATASETS” in ChEMBL 35) and so in future an effort to standardise deposited bioassay data will be undertaken as well. Recommendations have been provided to recent data depositors to increase the consistency of assay descriptions. As a response to depositor requirements, we have introduced new fields (ASSAY_GROUP) or populated the ASSAY_CATEGORY.

### Assay category

Inclusion of the ASSAY_CATEGORY field in the ASSAYS table originally allowed annotation of deposited PubChem assays with the bioassay type used by the depositor (“confirmatory”, “panel”, “screening”, “summary”, “other”). A depositor-defined ASSAY_CATEGORY allows grouping of assays as the depositor intended, in a more granular fashion than by ASSAY_TYPE. More recently, this field has been used within the EUbOPEN project [31] to organise depositions into categories such as “cell health”, “biochemical affinity” and “on-target cellular” to enable better understanding of the rationale behind the extraction of these assays from the literature to supplement chemical probe data. This metadata has also proven useful where depositors need specific categories to allow visualisation of data on bespoke web interfaces.

### Assay group

Comparison of assays across institutions is problematic. It is challenging for data users to determine whether individual assays have been performed in a truly comparable way and that all relevant metadata for assay interpretation has been captured by the depositor or author. Therefore, assays with the same compound-target pair may differ significantly in their read-out leading to challenges in interpretation.

The ASSAY_GROUP field was introduced to address this issue and enable more robust pooling of assays. Where a researcher or depositor has repeated an assay for different submitted datasets, or used the same commercial screening kit across different depositions, they are now able to flag these comparable assays using the ASSAY_GROUP flag. For data provenance reasons, these assays are still considered distinct and will have their own identifiers but they can be linked using the ASSAY_GROUP allowing users to choose whether to consider the assay set-up identical.

This field has so far been used by the AI-driven Structure-enabled Antiviral Platform (ASAP). For example, the same assay has been used across multiple depositions with both assays assigned to the same ASSAY_GROUP (“Human liver microsomal stability by Bienta—v1”). The first assays assessed 85 compounds for ChEMBL 35, and the same assay set-up was employed again to test 123 compounds for ChEMBL 36. Whilst the assay IDs are distinct for time-stamping and data provenance purposes, the assays can be considered identical as flagged by the same ASSAY_GROUP label.

### Curation of the assay organism

Assays within ChEMBL are annotated with an assay organism (in the ASSAYS.ASSAY_ORGANISM field). This is the organism used in the assay which may differ from the target organism. For example, a human protein expressed within a mouse model will be mapped to the human protein target but the assay organism should be recorded as mouse. Annotation of the assay organism provides an additional layer of detail that is valuable for interpretation and comparison of experimental set-ups.

The ASSAY_ORGANISM is a free-text field populated during data extraction or deposition and is provided alongside the numerical taxonomy ID (in ASSAYS.ASSAY_TAX_ID), sourced from the NCBI taxonomy [32]. Due to the free-text nature of the ASSAY_ORGANISM field, it is prone to variation due to misspelling, use of synonyms and updates to organism names and taxonomy as the NCBI classification evolves. Legacy organism data was out-of-sync with our primary organism ontology (NCBI taxonomy), adding complexity to data extraction pipelines and hindering comparison across assays. Due to renewed focus on assay curation, organism-related data contained within the ASSAYS table was updated for ChEMBL 34. Overall, alignment of organism names and taxonomy with the NCBI was improved and obsolete taxonomy IDs were replaced. In addition, where multiple strain level organism targets existed within ChEMBL, these were merged into the species target and the strain details migrated to the ASSAYS.ASSAY_STRAIN field. The advantage of merging organisms at the species level is that this avoids the need for a distinct target for each strain/isolate which splits data across many targets. Nevertheless, users should also consider the data alongside the strain-level information since this can have a profound impact on bioactivity. In this initial round of curation for ChEMBL 34, the ASSAY_ORGANISM/ASSAY_TAX_ID fields were updated for ~ 37 K legacy assays (with 60 obsolete assay tax IDs removed from the ASSAYS table). The organism details in the ASSAYS table are linked to the ORGANISM_CLASS table (our dictionary for organisms) and the TARGET_DICTIONARY (our dictionary of ChEMBL targets). Consequently, organism updates in the TARGET_DICTIONARY table affected 439 legacy rows and, in the ORGANISM_CLASS table, 72 updates to legacy data were made, with 25 entries downgraded. This round of updates prioritised the whole organism rather than protein targets, which require additional curation. Ongoing refinement over upcoming releases and routine alignment of incoming data with the NCBI will enable more robust and consistent annotation and simplify data extraction and analyses pipelines. Overall, these improvements to organism data provide valuable information for mapping assays to appropriate targets and model systems.

There is the potential for further enhancements in future around complex assay set-ups. For example, the ASSAY_ORGANISM/target ORGANISM fields cannot accurately capture cases where three organisms are relevant to an assay, e.g., “Antitumor activity against human HCT-116 cells expressing NfsA of Escherichia coli xenografted in NIH-III nude mouse assessed as tumor growth inhibition at 1000 micromol/kg, ip BID”. Similarly, where a viral target is expressed within a mammalian cell line, the assay organism is mammalian and the assay strain left as null leaving the only option to capture the strain as free text in the description. Future enhancements will also consider protein target organisms and extraction of strain data from assay descriptions.

### Protein variant curation

Protein variation can have a substantial impact on bioactivity data measurements for compound-target pairs. Furthermore, indels, isoforms, truncations and post-translational modifications can also impact protein activity and stability and should be considered during analysis of bioactivity data. An ideal scenario would include the publication and extraction of catalogue numbers for commercial proteins, or sequences for in-house expression constructs to avoid incorrect assumptions that the full-length, unmodified, wild-type sequence has been tested.

ChEMBL has included available information on protein variants in assay DESCRIPTONs since its inception. In version 22, substitution variants were specifically flagged in the ASSAYS table and annotated in the VARIANT_SEQUENCES table enabling users to more easily identify experiments performed with variant protein targets. A variant curation effort for ChEMBL 28 captured additional variants within legacy data and introduced a flag for undefined variants (such as those with deletions, insertions, or undefined changes from the wild-type sequence). A further curation push is planned to curate the remaining variants (substitution and non-substitution variants) and flag ambiguous cases.

**Fig. 3:**
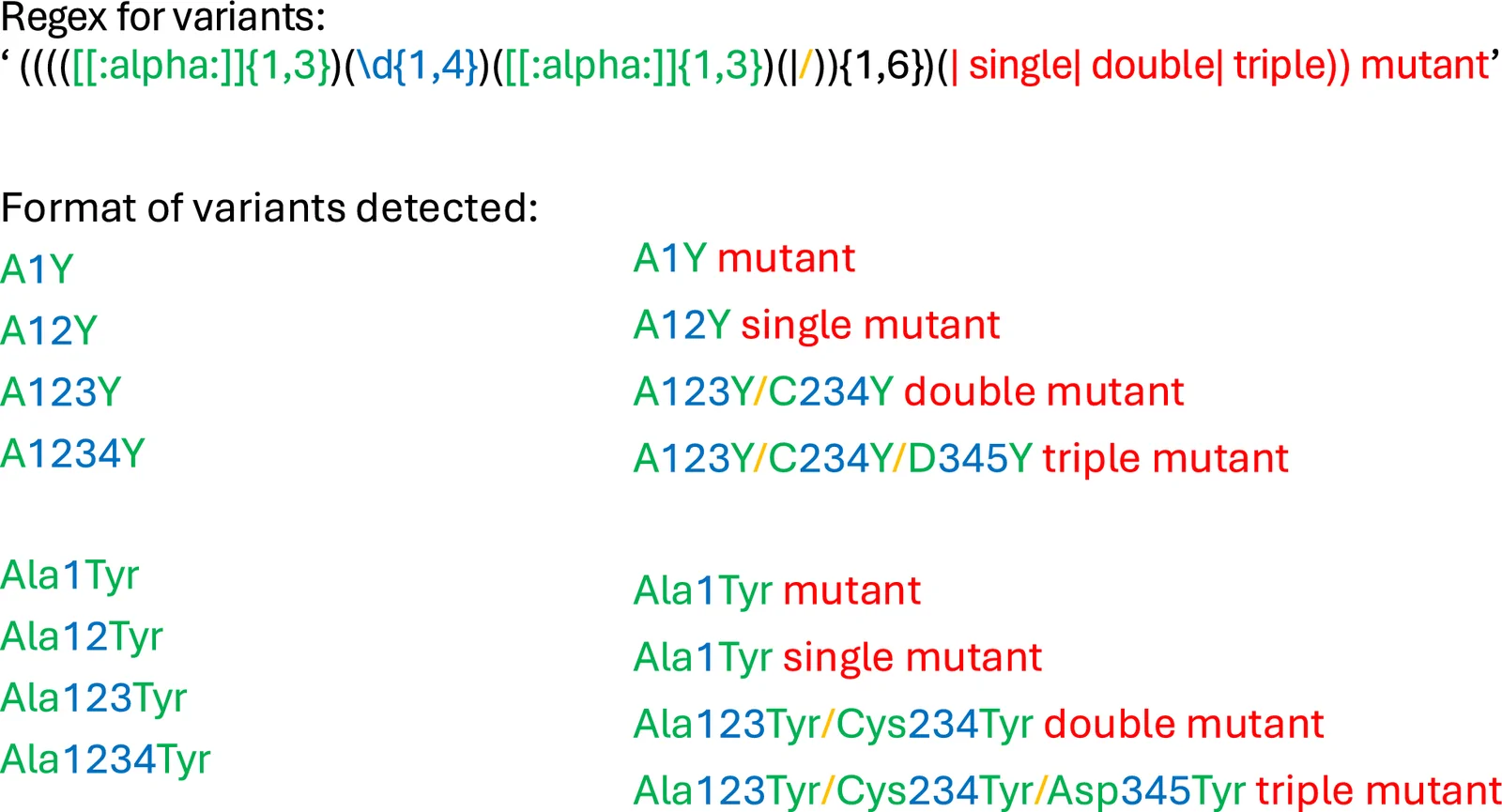
RegEx matching to substitution variants and the typical format of matched variants. Colour key: Green—amino acid (first entry is the parent amino acid and the second entry is the amino acid substitution); Blue—position of amino acid substitution; Red—additional qualifying text flagging the target as mutated

The current variant curation pipeline proceeds in two steps. First, variants are extracted from the core literature by our data extractors. Second, ChEMBL extracts and maps substitution variants using an internal target field and a regular expression (RegEx) that captures the preferred format for these sequence variations (Fig. 3). Other atypical variants (e.g., unspecified variants, deletions) provided by our data depositor are assigned an unclassified (-1) flag or manually reviewed. Datasets provided by independent data depositors are manually curated in discussion with depositors, but as deposited data provides an increasingly significant portion of ChEMBL, more stringent rules on the provision and format of variants is required.

Further curation effort for protein variants was made for ChEMBL 35 as part of a collaboration with Leiden University [33]. As a result, legacy and incoming data were reviewed and curated resulting in 2661 variants (188 new variants) with 17,070 assays (3293 newly annotated assays) mapped to variants and 107,683 activities (10,696 new activities) mapped to variants.

### Annotation improvement for ADME assays in ChEMBL

As discussed above, the way ChEMBL captures assay details has evolved over time. Therefore, new rules for data curation may not be straightforward to apply retrospectively on legacy data. For instance, the ASSAY_PARAMETERS table was introduced with ChEMBL 18 but at the time it contained assay metadata for the newly extracted data only. For the following releases of ChEMBL, the number of assays with metadata increased but only at the pace at which new data has been added.

To address this problem, we initiated a retrospective analysis on selected ADME assays in ChEMBL to see if—with the information originally extracted by the data extractors and stored in the assay description—we could enhance their re-usability. We focused on a subset of 17,797 assays related to two ADME endpoints frequently used for Pharmacokinetic/Pharmacodynamic (PK/PD) analyses comprising 11,075 AUC and 6702 C_max_ bioactivity data points. Comparing these data only makes sense when the associated parameters are the same. For instance, for the same dose, a C_max_ after an oral or intravenous administration is expected to be different as the intestinal absorption reduces the quantity of a measurable substance in the blood stream. The dose is also of high importance as a low or high dosage often result in different measurements.

Historically, these parameters were extracted by the data extractors alongside the actual C_max_ or AUC values, but because of a lack of a more suitable place to store them within the database, they have been recorded as part of the assay DESCRIPTIONs. Hence, it was possible to extract the relevant information from the assay DESCRIPTION using an extensive list of RegEx. For this study, we focused on the following PK/PD parameters: “dose” and “dose unit”, “route of administration”, and “time” (the latter for AUC only).

As shown in Table 2, the RegEx properly extracts the relevant information and the relevant parameter can later be standardised and stored in the ASSAY_PARAMETERS table. The code developed for this work is publicly available (see section “Availability of data and materials”).

**Table 2:** Examples for ChEMBL assay descriptions highlighting the syntactic similarity, allowing to capture PK/PD parameters

Assay ChEMBL ID	Description	Dose value	Dose unit	Route of administration	Time
CHEMBL1837348	Cmax in Wistar BRL rat plasma at 5 mg/kg, po	5	mg/kg	po	
CHEMBL2066310	AUC (0 to 4 h) in rat at 2 mg/kg, iv	2	mg/kg	iv	0 to 4 h
CHEMBL5055424	Cmax in Swiss albino mouse at 50 mg/kg, ip after 2 h by LC–MS/MS method	50	mg/kg	ip	
CHEMBL1660925	Cmax in Sprague–Dawley rat at 170 umol/kg, po qd measured on day 1	170	umol/kg	po	
CHEMBL3386393	AUC (0 to 6 h) in rat at 10 mg/kg, sc	10	mg/kg	sc	0 to 6 h

As validation, we compared the parameters extracted by the RegEx method with those manually extracted for the most recent ChEMBL data (from ChEMBL 24 onwards). A subset of 34,270 parameters was identified in which 99.9% of “dose”, “dose unit”, and “route of administration” terms agreed between the two approaches. For the “time” parameter, no comparable manually annotated terms were available in ASSAY_PARAMETERS at the time we ran the analysis.

In summary, deploying the RegEx approach for 17,797 assays with C_max_ and AUC endpoints, additional parameters were retrieved for a total of 11,261 and 19,661 bioactivity data points, respectively. In terms of ChEMBL bibliography, the additional parameters cover 2617 documents for C_max_, and 3,398 documents for AUC. This represents a significant enrichment of existing ChEMBL data with relevant assay parameters using automated data extraction from assay descriptions.

### Mapping endogenous cell lines to cell ontology

For cell-based assays, the corresponding assays are mapped to an entry in the CELL_DICTIONARY. To ensure FAIR data, the CELL_DICTIONARY is cross-referenced to relevant ontologies. For cell lines, several well established ontologies, such as Cellosaurus [21], are used. However, an ontology for primary cells was not present in the dictionary and therefore links to the Cell Ontology [22] were added for ChEMBL 26, increasing the interoperability of ChEMBL cell-level data.

### Approaches to enhance assay descriptions through AI-driven strategies

Understanding the interactions between compound-target pairs, as reflected in their bioactivity profile, requires a comprehensive understanding of the bioassay set-up. Models using the output from diverse assays can introduce unnecessary noise and confound the results. In ChEMBL, bioassays are recorded with a brief free-text description, and these could be used to improve the annotation and classification of assays and allow users to select appropriate inputs for generating predictive models. For this purpose, two strategies were considered; named entity recognition (NER) and a multi-class “broad classification” model. The NER approach focuses on the recognition of the experimental method or instrument used to measure an assay readout. The multi-class model aims to provide more granular assay categories compared to the existing ASSAY_TYPE (F, B, A, T, P, U). This could reduce the diversity of assays within a single category, for example the F (Functional) ASSAY_TYPE currently contains target-based activity assays, cell phenotype and organism-level assays. The two approaches are complementary since annotation of a method alone does not inform a user of the broad assay class and an assay class alone does not provide insights into the methodology; both the assay class and extracted experimental method entity have been linked to the Bioassay Ontology (BAO) ontology if possible.

### Annotating the experimental method using named entity recognition

**Fig. 4:**
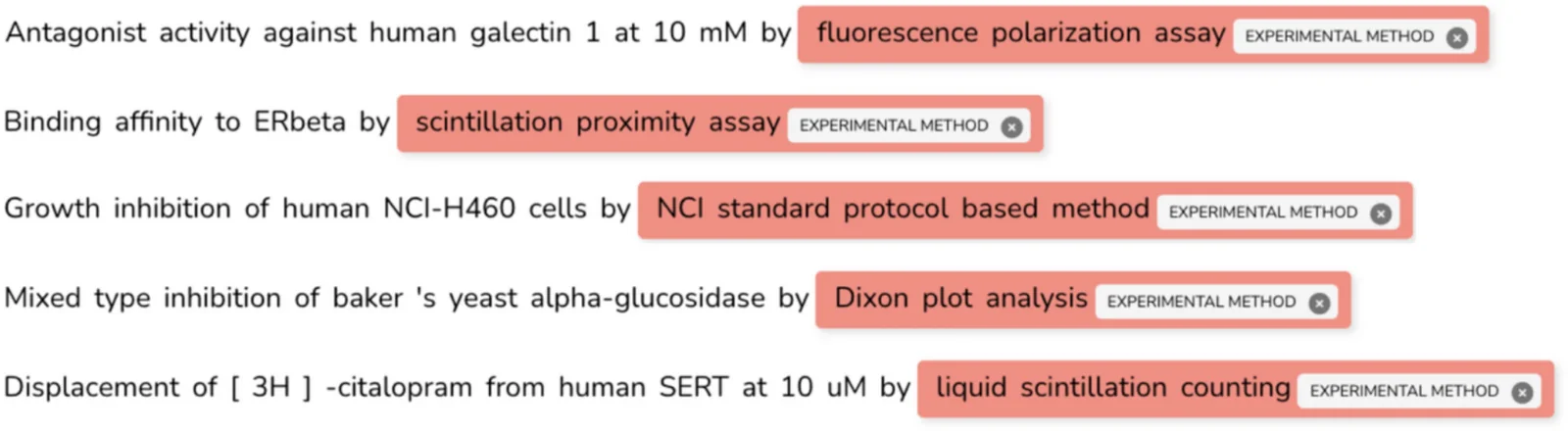
Illustrative examples of manually tagged experimental method terms for the training corpus of the NER model created by using the NER Annotation tool for spaCy [34]

To enable the identification of the experimental assay setup referred to as the “experimental method”—a specific tool, instrument, assay methodology, or kit used to measure the experimental read-out—within assay descriptions, a spaCy Named Entity Recognition (NER) model was developed. This model was trained using a manually annotated corpus of 800 assays, comprising 500 binding assays and 300 functional assays, where methodology-related terms were manually tagged. Of the 800 assays, 460 had annotated methods, while 340 lacked any mention of methodology. Among the annotated method terms, 272 were unique (considering string variations). Some illustrative examples of manually tagged experimental method terms can be found in Fig. 4 and a detailed description of the manual annotation process can be found in the “Methods” section.

**Fig. 5:**
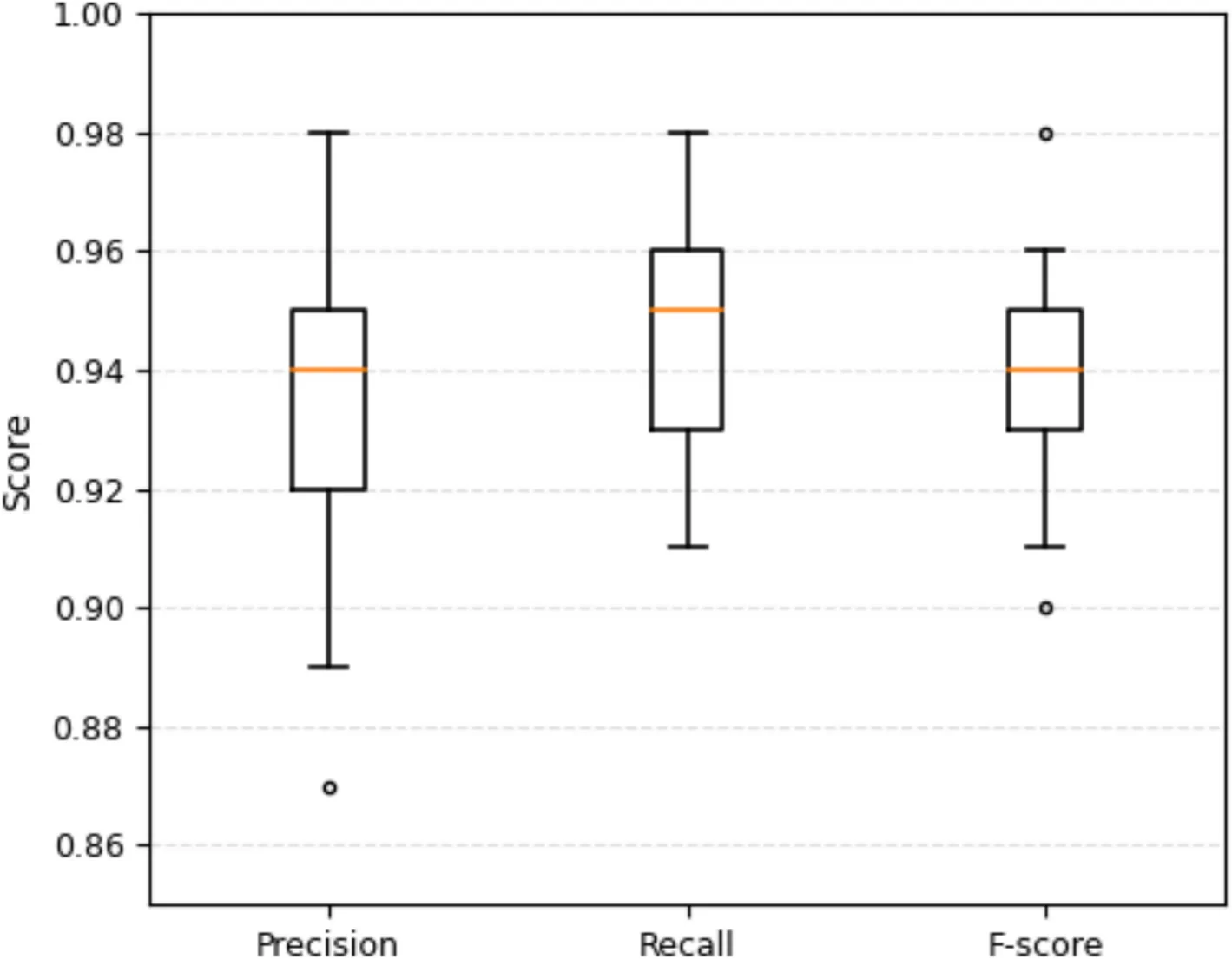
Cross Validation (CV) performance of the Named Entity Recognition (NER) model for predicting the “experimental method” terms

First, the NER model was trained using the annotated data to predict the experimental method described within a given assay description. To robustly evaluate the model's performance, a repeated fivefold cross-validation was employed. This process involved randomly partitioning the data into five folds, training the model on four folds, and testing on the remaining fold. This entire fivefold cross-validation was repeated five times, each with a new random split of the data, resulting in a total of 25 training and testing cycles. The performance across these 25 folds yielded average precision, recall, and F1-score values of 0.93, 0.95, and 0.94, respectively (Fig. 5; Supplementary Table S2).

Following the strong performance observed during cross-validation, a final model was trained using the complete dataset (800 assays). This model was then applied to identify the assay method within 1,169,293 B-type and F-type assay descriptions in ChEMBL 35 derived from source ID 1 (“Scientific Literature”) and with a curated target (excluding targets with target_id 22226). Of these, 392,592 (33.6%) corresponded to B-type and 776,701 (66.4%) to F-type assays.

The NER model execution yielded a total of 662,675 assays where the model was able to identify an experimental method which corresponds to 57% of all B- and F-type assays and 38% of all assays in ChEMBL 35. The model identified 44,185 unique terms for the experimental method annotation. The most frequently identified methods included “MTT assay”, “sulforhodamine B assay”, “Western blot analysis”, “SRB assay”, “broth microdilution method”, and “ELISA” among others. See Table 3 for the 20 most frequent methods or Supplementary Table S3 for the full list.

**Table 3:** The 20 most frequent Experimental Method terms found in B-type and F -type assay descriptions of ChEMBL 35 detected by the NER model

Method term (span)	Frequency (number of assays)
MTT assay	45,793
Sulforhodamine B assay	25,249
Western blot analysis	24,214
SRB assay	23,681
Broth microdilution method	14,574
ELISA	10,705
Kinomescan method	8830
Fluorescence assay	6218
Agar dilution method	6179
Flow cytometry	5866
Scintillation counting	5195
Propidium iodide staining based flow cytometry	5079
Luciferase reporter gene assay	4745
MTS assay	4634
Scintillation counting method	4452
Liquid scintillation counting	3913
Western blotting analysis	3142
HTRF assay	3009
TR-FRET assay	2985
Spectrophotometry	2979

To assess the accuracy of the model's entity recognition on external data, a stratified random sample of 200 assays with annotated (predicted) experimental method by the NER model was selected, comprising 68 B-type and 132 F-type assays. The sample also included assay descriptions without an annotated method. The assays were manually reviewed, labelling them according to the model's performance (for methodological details see “Methods” section).

**Fig. 6:**
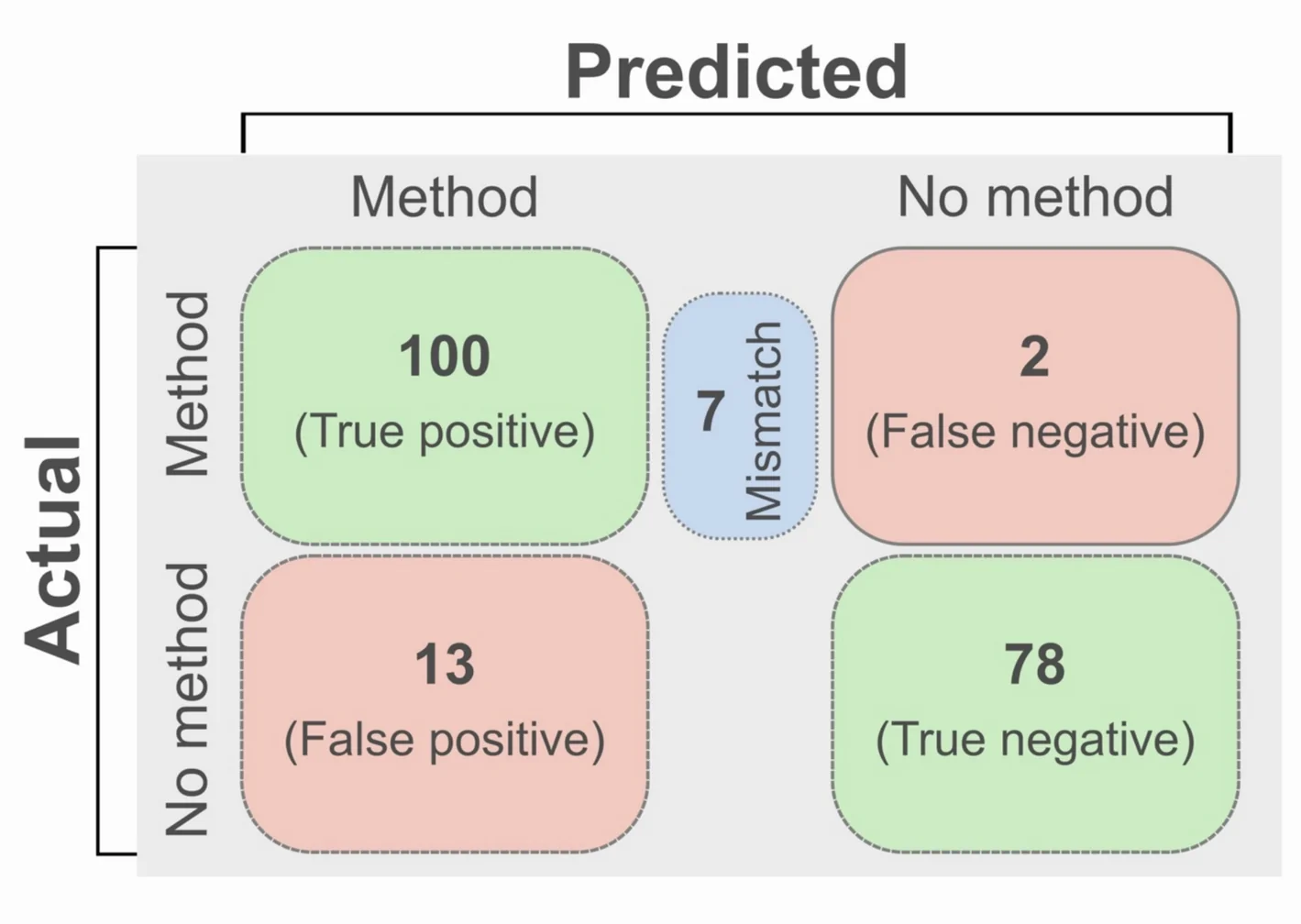
Confusion matrix evaluating the NER model. ChEMBL curator annotations (rows) are compared to NER model predictions (columns). Method refers to when an assay method or kit has been identified either by the model or by curators and no method refers to assay descriptions without an identified method. “Mismatch” is for cases where a method has been identified by the model, but it differs from the annotated method by a few tokens

The confusion matrix in Fig. 6 demonstrated the model's robust performance in identifying methods within assay descriptions. The model achieved a high true positive count of 100, with a low false negative count of 2, indicating effective identification of the majority of methods. While 13 false positives were observed, suggesting some instances of incorrect method identification, the low false negative count suggests strong recall. Mismatches, numbering 7, indicate the model’s ability to identify portions of method names, highlighting potential areas for further refinement. A true negative count of 78 further confirms the model's ability to correctly identify text where methods were absent.

Analysis of mismatched method labels revealed several distinct categories. These included: full mismatches, where the predicted and annotated methods shared no tokens; partial matches, characterized by differing numbers of tokens between the predicted and annotated methods; split annotations, where a single annotated method was predicted as two separate methods; and multi-prediction, in which the model predicted two methods, only one of which corresponded to the annotation (Table 4).

**Table 4:** Assay descriptions with a mismatch between the predicted method by the NER model and annotated method. The column Mismatch type indicates the cause of mismatching between annotated and predicted methods

ChEMBL Assay ID	ASSAY_TYPE	DESCRIPTION	Predicted method	Annotated method	Mismatch type
CHEMBL4337148	B	Inhibition of recombinant human TRKC (510 to end residues) assessed as residual activity at 10 uM using GEEPLYWSFPAKK as substrate incubated for 40 min in presence of [gamma33P]ATP by scintillation counting based radiometry assay relative to control	Scintillation counting	Scintillation counting based radiometry assay	Partial match
CHEMBL5149189	B	Inhibition of full length mouse recombinant MoPrP (23 to 231 residues) aggregation in RML prion infected mouse ScN2a cell lysate using PrPSc as substrate by Thioflavin T based fluorometric assay	Fluorometric assay	Thioflavin T based fluorometric assay	Partial match
CHEMBL5244462	B	Binding affinity to human recombinant ALK L1196M mutant (1093 to 1411 residues) assessed as inhibition constant using 5'FAM-KKSRGDYMTMQIG-CONH2 peptide as substrate in presence of ATP by micro-fluidic mobility shift assay	Inhibition constant	Micro-fluidic mobility shift assay	Full mismatch
CHEMBL4252837	B	Inhibition of human DNA topoisomerase 2 alpha-mediated DNA relaxation at 20 uM incubated for 30 min using using pBR322 DNA substrate by ethidium bromide dye-based agarose gel electrophoresis	Ethidium bromide dye-based-agarose gel electrophoresis	Ethidium bromide dye-based agarose gel electrophoresis	Split term
CHEMBL4015161	B	Inhibition of human URAT1 expressed in HEK293T cells assessed as reduction in [14C]uric acid uptake at 30 uM after 5 min by liquid scintillation counting method relative to control	Liquid scintillation counting	Liquid scintillation counting method	Partial match
CHEMBL4121829	F	Induction of apoptotic nuclear morphological changes in human MCF7 cells assessed as induction of DNA fragmentation at 12 uM incubated for 48 h by DAPI nuclear staining based fluorescence microscopy	Fluorescence microscopy	DAPI nuclear staining based fluorescence microscopy	Partial match
CHEMBL4811144	B	Inhibition of equine serum BChE using butyrylthiocholine iodide as substrate preincubated with enzyme for 30 min followed by substrate addition and measured after 30 min by Ellman's method	Butyrylthiocholine iodide as substrate preincubated with enzyme for 30 min followed-Ellman’s method	Ellman’s method	Multi prediction: one correct term and one full mismatch

### Linking experimental method terms to bioassay ontology

The same 800 assays that were used for training the NER model for detecting the experimental method also served as a gold standard data set for BAO linking: the extracted strings were manually annotated with ontological terms in BAO, where possible. Out of these, 460 assay descriptions (57.5%) contained a mention of an “experimental method”, and for 407 of these, an appropriate BAO term could be identified. 93% of these assays (377 assays) were annotated with a single BAO term, whereas for 19 assays two BAO terms were necessary to comprehensively describe the experimental method. Another 11 assays, even required three BAO terms to fully map the entities in the description. We identified 225 unique text strings describing the experimental method in the gold standard data set which are linked to 83 unique BAO terms.

Next, the text2term ontology annotation tool [35] was used to suggest terms for the gold standard data set which were subsequently compared with the manually annotated terms. Each suggestion from text2term is supported by a mapping score, as shown by the examples in Table 5.

**Table 5:** Examples of BAO term mappings with the text2term tool vs manual annotation: CHEMBL4230871 shows an example of an assay with one BAO term annotated in the gold standard data set, which was correctly identified with the highest mapping score

Assay description [ChEMBL Assay ID]	Extracted experimental method term	Gold standard—BAO preferred term	Mapped BAO term	Mapped BAO ID	Mapping score	Evaluation
Binding affinity to wild-type human full length PHKG1 (M1 to Y387 residues) expressed in bacterial expression system assessed as residual binding level at 1 uM by Kinomescan method relative to control [CHEMBL4230871]	Kinomescan method	KINOMEscan assay	KINOMEscan assay	BAO_0003011	0.81	Correct
s.a.	s.a.	s.a.	kinase active-site binding method	BAO_0003030	0.329	Incorrect
s.a.	s.a.	s.a.	assay design method	BAO_0002202	0.308	Incorrect
s.a.	s.a.	s.a.	MES	BAO_0002720	0.291	Incorrect
s.a.	s.a.	s.a.	S phase assessment method	BAO_0002740	0.257	Incorrect
Induction of apoptosis in human HCC827 cells assessed as increase in hypodiploid DNA content at 50 to 100 nM after 72 h by propidium iodide staining flow cytometry [CHEMBL4142897]	Propidium iodide staining flow cytometry	Flow cytometry	flow cytometry	BAO_0000005	0.609	Correct
s.a.	s.a.	Propidium iodide DNA staining	Not suggested	N/A		Not suggested (false negative)
s.a.	s.a.	s.a.	Gram staining	BAO_0002844	0.296	Incorrect
s.a.	s.a.	s.a.	mass spectrometry	BAO_0000055	0.212	Incorrect
s.a.	s.a.	s.a.	cytosol stability assay	BAO_0010096	0.183	Incorrect

s.a.: see above

CHEMBL4142897 shows an example of an assay with two annotated BAO terms, of which only one could be retrieved by text2term

Reassuringly, we observed a good separation between correct vs incorrect terms in the gold standard data set as evaluated by the mapping scores (Supplementary Figure S1), showing that the score is a useful proxy for assessing the confidence of the mappings. Next, precision and recall were evaluated at different score cut offs (Supplementary Figure S2). For example, considering all suggestions with a score higher than 0.7 gave a precision of 0.75 (3 in 4 suggestions are correct) but a recall of only 0.15 (15% of the BAO terms in the gold standard are retrieved). This shows that while most of the resulting mappings are expected to be correct, only a small fraction of the possible BAO terms could be mapped by the tool, clearly showing the limitations of the method.

Finally, all B- and F-type assays from ChEMBL 35 derived from scientific literature (src_id 1) with a defined target for which the NER model identified an “experimental method” entity were submitted for BAO annotation with the text2term tool. Out of these 1.17 M assays, for 662,675 (57%) an “experimental method” could be annotated by the NER model, comprising 44,185 unique method strings. Of these, 52% could be annotated with at least one BAO term with a mapping score > 0.3 and 14% with a mapping score > 0.6. At a minimum mapping score of 0.6, a total of ~ 8% of all assays in ChEMBL 35 have at least one BAO annotation, with a total of 395 unique BAO terms assigned to 4150 unique “experimental method” terms across this set.

In future work, we will continue to explore different methods for ontology linking/concept normalisation, both using advanced NLP techniques beyond text2term (such as neural transformer-based methods) as well as simpler dictionary-based approaches. Manually annotating the ~ 2800 most frequent method terms would allow for coverage of approximately 80% of the assays in ChEMBL (Supplementary Figure S3). Thus, we plan to explore a hybrid model, using a core dictionary for the most frequent terms and an automated approach for the less frequent ones.

### Complementing ASSAY_TYPE annotations in ChEMBL: classification model for broad assay aim category

To complement the current ASSAY_TYPE in ChEMBL we aimed to develop an assay categorisation that should capture the type of the assay in a more granular and hierarchical manner. It should also be complementary to the existing BAO_FORMAT annotations within ChEMBL, which specify the primary assay entity (i.e., “cell-based”, “organism-based”). For example, a target-based cellular assay and a cytotoxicity assay have the same BAO-format (“cell-based format”) but very distinct assay aims. The newly developed broad assay category offers a means to separate these assays by overall assay setup or assay aim and, for example, offer the means to distinguish cytotoxicity assays from other cell-based assays.

Therefore, we manually annotated 900 B- and F-type assays with the broad assay aim category in order to be used to train a multi-label classification model. The resulting broad categories across these assays, their assay counts, mapped BAO terms (if available) and an exemplar assay description, respectively, are shown in Table 6.

**Table 6:** Broad assay categories annotated in a set of 900 B and F assays with corresponding BAO mappings and an example of a representative assay description, respectively. The assay aim categories highlighted in bold have been used for model training

Assay aim category	Assay count	BioAssay Ontology preferred term	BAO ID	Exemplar assay description [Assay ID]
Protein activity	171	Functional target-based	BAO_0013016	Agonist activity at human RXR-alpha expressed in U2OS cells assessed as maximum relative efficacy at 100 uM by PathHunter assay relative to 9-cis-retinoic acid [2235577]
Binding	134	Binding assay	BAO_0002989	Binding affinity to human MAP3K2 at 1 uM by KINOMEscan scanMAX assay relative to control [2122060]
Radioligand binding	118	Radioligand binding assay	BAO_0002776	Displacement of [3H]-SCH23390 from D3 receptor (unknown origin) expressed in HEK293T cell membranes measured after 2 h by microbeta scintillation counting method [2132583]
Cell phenotype	113	Cell phenotype	BAO_0002542	Cytotoxicity against human Ramos cells assessed as cell death incubated at 10 uM for 20 h measured by DNS assay [2154913]
Antimicrobial activity	88	–	No suitable ID	Antibacterial activity against Arcobacter butzleri after 17 h by NCCLS broth microdilution method [1795430]
In vivo method	63	In vivo assay method	BAO_0040021	Antiinflammatory activity in Wistar albino rat assessed as reduction in carrageenan-induced paw volume at 12.5 mg/kg, ip measured after 1 h by plethysmometric method (Rvb = 1.43 ± 0.19 ml) [1862272]
Nucleic acid binding	26 + 150*	–	No suitable ID	Ability to interact with DNA was assessed by a DNA/ethidium bromide dye displacement assay, expressed as DC50 for polydAdT [52456]
Unclear	20			Binding affinity towards rat kidney Vasopressin V2 receptor by using functional assay [215016]
Whole-organism assay	6			Induction of ultrastructure damage of root tip statocytes in Zea mays (maize) seedlings assessed as reduction in organelle size at 400 ug/L measured 48 h post dose by transmission electron microscopy [894707]
Antioxidant activity	5			Antioxidant activity assessed as DPPH radical scavenging activity at 40 uM after 30 min [976258]
Binding/activity—mixed measurements	2			Evaluated for its agonist activity and the binding affinity against human Beta-1 adrenergic receptor in membranes from chinese hamster ovary cell [37378]
Prodrug metabolism	1			NO releasing activity assessed as NO release efficiency at 100 uM by Griess assay relative to NaNO2 [2156446]
Protein–protein interaction assay	1			Inhibition of C-terminal 6-His-tagged beta-catenin (1 to 781 residues) (unknown origin) expressed in Escherichia coli BL21 (DE3)-N-terminal biotinylated human BCL9 (350 to 375 residues) protein–protein interaction incubated for 1 h by Alphascreen assay [2131421]
Stimulation of binding	1			Ability to stimulate binding to delta-opioid receptor using GTP-gamma -S-binding assay in guinea pig caudate membranes, blocked with 20 nM naltrindole (NTI); NS = no stimulation [147295]
Tissue-based assay	1			Anti-urolithic activity in synthetic urine assessed as decrease in absorbance at 10 mg/mL relative to control [1987633]

^*^In the first pass, there were only 26 instances in the “Nucleic acid binding” category which was at the edge of our cutoff for considering specific categories (20 instances). We therefore subsequently supplemented this category with another 150 assays to ensure sufficient instances to be able to include this category for modelling

For the seven largest assay aim categories (“Protein Activity”, “Binding”, Radioligand Binding”, “Cell Phenotype”, “Antimicrobial Activity”, “In Vivo Method”, and “Nucleic Acid Binding”) with sufficient training data available (> 50 assays), a multi-class classification model was trained using spaCy to predict these categories for a given assay description. The average precision, recall and F1-score of the models during cross-validation are shown in Table 7 and the detailed performances per fold are visualised in Supplementary Figure S4. The models show very good performance with the lowest average performance being a recall of 0.79 for the protein activity category.

**Table 7:** Average precision, recall and F1-score of the models during repeated fivefold cross validation (“CV”) as well as on an independent test set (“Eval”)

Assay category	CV: precision	CV: recall	CV: F1-score	Eval: precision	Eval: recall	Eval: F1-score	Annotated assays (Eval)
Protein activity	0.84	0.79	0.81	0.80	0.90	0.85	46
Cell phenotype	0.88	0.83	0.85	0.95	0.93	0.94	43
Antimicrobial activity	0.95	0.83	0.88	0.82	0.97	0.89	42
In vivo method	0.97	0.87	0.91	0.88	0.84	0.86	29
Binding	0.89	0.85	0.87	0.92	1.00	0.96	11
Radioligand binding	0.95	0.91	0.93	1.00	1.00	1.00	10
Nucleic acid binding	0.96	0.94	0.95	0.00	0.00	0.00	0

**Fig. 7:**
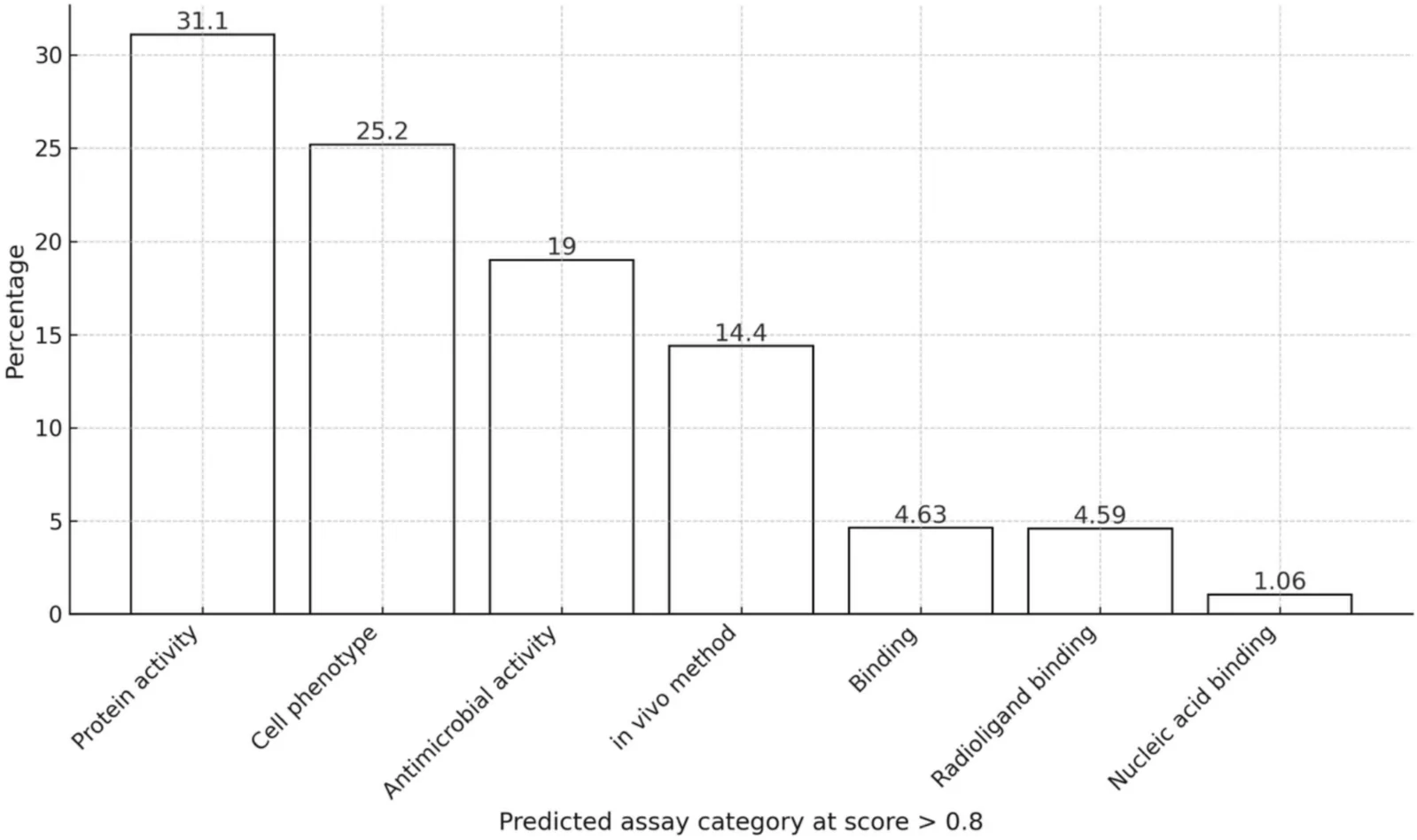
Distribution of predicted broad assay aim categories for B-type and F-type assays in ChEMBL 35 for predictions with a prediction score > 0.8

Next, we used the model to predict the broad assay aim categories for B- and F-type assays in ChEMBL 35 derived from scientific literature (src_id 1) and with a defined target (TID ! = 22226). We found that 88% of these assays have a prediction with a prediction score > 0.8 for one assay aim category (Table 8). At this prediction score, 86% of the F-type assays are annotated with an assay category and 91% of the B-type assays. The distribution of the resulting broad assay aim category predictions is shown in Fig. 7, showing that the categories “Protein Activity” and “Cell Phenotype” are the largest predicted assay categories. It has to be noted, however, that some categories represent higher level categories (e.g., “Protein activity”) while others could be seen as sub-categories (e.g., “Radiologand Binding” is a sub-category of “Binding”) which might also explain lower numbers of representatives for some of the categories.

**Table 8:** Counts of B- and F-type assays in ChEMBL 35 that have predictions from the broad assay aim category model at different prediction scores

Prediction score	Number of assays annotated	Percentage of assays annotated	Percentage of F-type assays annotated	Percentage of B-type assays annotated
> 0.8	1,026,488	88	86	91
> 0.7	1,047,718	90	88	93
> 0.5	1,080,280	92	91	95

Additionally, we evaluated the predictions of the broad assay category model for ChEMBL 35 assays on an independent validation set of 200 randomly selected B- and F-type assays. When the assay category with the highest prediction score (> 0.8) was chosen, the predictions showed 81% agreement with the manually chosen assay category. The agreement increased to 86% when considering predictions with a minimum prediction score > 0.7.

**Fig. 8:**
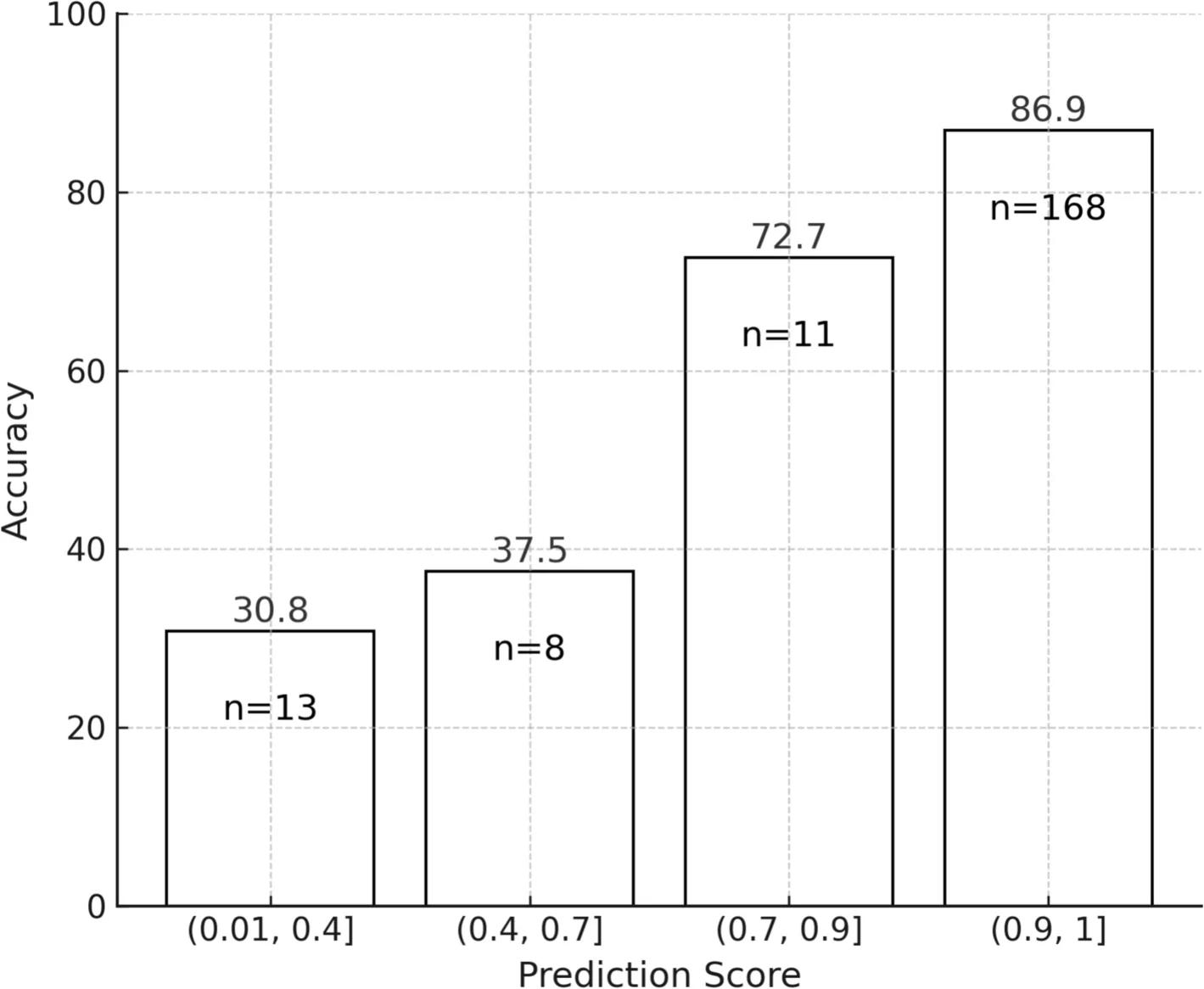
Accuracy when choosing different prediction scores assessed by an external validation set of 200 manually annotated randomly selected B- and F-type assays. The numbers printed on the bars indicate the number of assays that have their maximum prediction score within the indicated range. The numbers on top of the bars show the accuracies per bin (percentage of assays correctly predicted in each bin)

To explore the value of setting a higher cut off for the prediction score, we assessed the agreement with manual annotations for different bins of the prediction threshold (Fig. 8). This confirmed that the accuracy is highest (86.9%) for prediction scores between 0.9 and 1. In contrast, for lower prediction scores (below 0.7) the accuracy drops to below 40%. Thus, the prediction score appears to correlate with accuracy of the predictions and gives confidence in using a higher cut-off for the prediction score when deploying the model on unseen data.

The precision and recall when predicting the external validation set for the different assay categories is shown in Table 7. For this evaluation, the predicted category for each assay was taken as the assay category with the highest prediction score output by the multiclass model. Overall, the performance on the external validation set is similar to the performance observed during cross-validation except for the “nucleic acid binding” category. Two assays were predicted with the category “nucleic acid binding” for the validation set, but these were both incorrect predictions (as there were no assays annotated with this category in the validation set). As such, the small size of this category makes it hard to assess performance using the validation set, and so we decided to examine another 50 assays predicted as nucleic acid binding assays to better understand the performance.

We randomly selected 50 assays from ChEMBL 35 that were predicted as “nucleic acid binding” assays with any prediction score, excluding any assays used for training. These predictions were then manually reviewed and marked as correct or incorrect. When considering the predictions with a prediction score > 0.7, 63% of the predictions were correct, while none of the predictions with a score < 0.7 were correct. This performance shows a drop compared to the cross-validation performance, where precision and recall were both higher than 0.94. One possible explanation is that due to the small size of this category, the training assays were supplemented with manually selected assays, rather than derived from random sampling, and we believe this could have resulted in a rather similar set of assays being used for training. It appears that the model struggles to extend well to the variety of assays in ChEMBL. However, due to this being a small category (~ 1% of assays are predicted as this category), it does not greatly affect overall performance. In addition, it was reassuring that the prediction score appeared to correlate with the accuracy such that choosing a higher prediction score can still provide reasonable predictions.

## Discussion

In this comprehensive study, we have addressed the challenge of annotating legacy data by demonstrating the feasibility of retrospective enrichment of assay metadata. Using a combination of machine learning (ML), natural language processing (NLP), and rule-based approaches (e.g., regular expressions), we successfully extracted ADME-relevant parameters and other key metadata from unstructured assay descriptions. This has enabled the recovery of crucial experimental details, unlocking new potential for robust large-scale QSAR modeling.

ChEMBL's existing annotation framework—ASSAY_TYPE and BAO_FORMAT—captures high-level experimental setups and primary biological systems, respectively. Our work builds on this foundation by introducing two complementary, modular components:

1. A structured annotation of the experimental method employed.
2. A broad assay aim category that describes the intent and design of the assay.

A key contribution was the implementation of a Named Entity Recognition (NER) model for large-scale extraction of "Experimental Method" terms from free-text descriptions. The model achieved high precision and recall, supporting finer-grained annotation and improving assay interpretability for end-users. In parallel, we developed a broad assay aim classification model, which introduces a more nuanced categorization layer beyond the existing high-level ASSAY_TYPE labels (e.g., "Binding", "Functional"). This allows for the disambiguation of biologically similar but functionally distinct assays, supporting more informed data selection for cheminformatics analyses and predictive modeling.

As the assay annotation system stands now, it shall be used in a modular way combining the different systems of assay categorisation in order to identify the assays that use an identical assay format, follow the same broad assay aim and use the same experimental method.

**Fig. 9:**
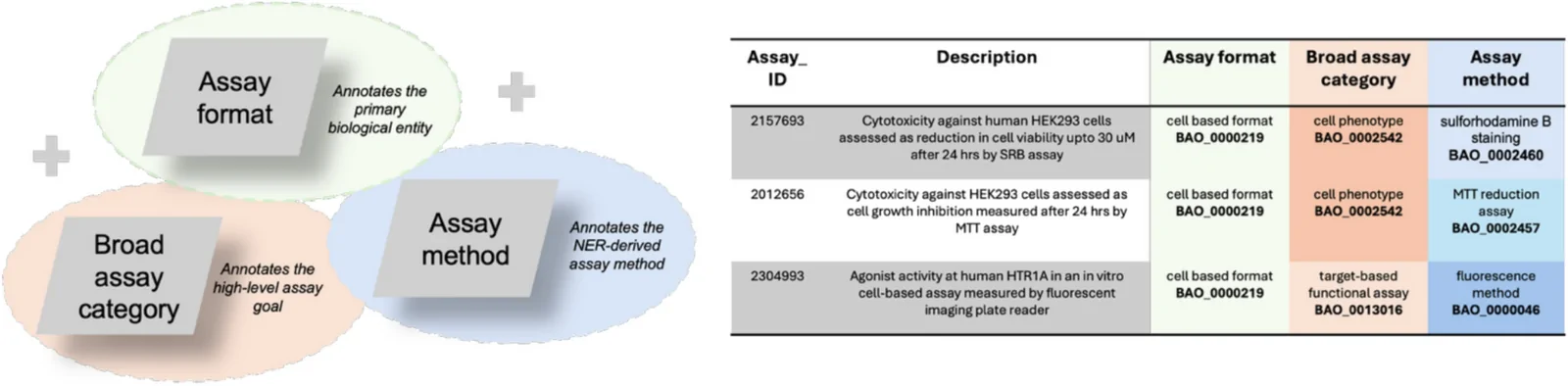
Modularity of the new assay annotation system. The annotation components “Assay format”, “Broad assay category”, and “Assay method” can be used in conjunction to enable a user to select assays with identical assay format, the same broad assay aim, and identical assay methods

For instance, a target-based cellular assay and a cytotoxicity assay may both be labeled under the same BAO format (e.g., cell-based) yet pursue very different objectives. Our expanded classification scheme enables these distinctions, offering more targeted and biologically meaningful grouping of assays. Figure 9 shows three examples of assays in ChEMBL which differ on the level of the broad assay aim and the experimental method used.

The new bioassay annotations have been made available on the ChEMBL FTP server [36]. For future releases of ChEMBL we plan to integrate the annotations into the ChEMBL database schema and to update the new incoming data as part of the release process, including a regular update of ontological mappings to BAO.

Overall, our ontology alignment efforts—including mapping to BAO terms and other ontologies and refining organism/protein variant annotations—strengthen ChEMBL’s adherence to FAIR data principles and facilitate deeper integration with external biomedical resources.

Looking ahead, we plan to:

- Extend these annotation strategies to additional ASSAY_TYPE categories.
- Refine our NLP models through active learning.
- Explore both encoder-style transformer models and GPT-like LLMs for bioassay classification.
- Deepen integration with BAO by contributing missing terms to BAO or extending mappings to other ontologies.
- Evaluate hybrid strategies for ontological linking, combining dictionary-based and automated methods.

Close collaboration with data extractors, data depositors, and the broader community will remain essential to promoting best practices in assay description and metadata deposition. These ongoing efforts aim to make ChEMBL’s bioassay data more richly annotated, AI-ready, and maximally useful for downstream applications.

## Conclusions

This study highlights significant advancements in the annotation and classification of bioassays within the ChEMBL database, driven by a combination of manual curation efforts and the integration of AI-based methodologies. Through the development of a structured assay description framework and the application of natural language processing (NLP)/ML tools, we demonstrate how both the interpretability and reusability of bioactivity data can be substantially improved.

In conclusion, by combining expert domain knowledge with scalable AI methodologies, we provide a blueprint for transforming unstructured experimental descriptions into richly annotated, interoperable data. These developments represent a major step forward in making bioactivity data more actionable (re-usable), re-producible, and aligned with modern data science workflows.

## Materials and methods

### Extraction of PK/PD parameters using RegEx

Bioactivity measurements with C_max_ and AUC STANDARD_TYPE in the ACTIVITIES table were selected from ChEMBL 33 along with their related assay DESCRIPTION from the ASSAYS table. Different regular expression (RegEx) strings were used for the different PK/PD parameters: “dose”, “dose unit”, “route of administration” and “time” (the latter only for the AUC activities). The RegEx strings were elaborated during an iterative process. For instance, the RegEx string for extracting the “dose” and “unit” was first designed to capture a digit followed by a space character followed by “mg/kg” (e.g., “10 mg/kg” as in CHEMBL620322—“max value after oral dose at a dose of 10 mg/kg in rats.”). The corresponding RegEx string is “\d + mg/kg”. However, we found multiple different ways of noting down the same “dose” expression, changing only one character. For example, the space character between the “dose” value and the “unit” might be missing (e.g., “10 mg/kg” as in CHEMBL906216—“AUC (0–24 h) in Sprague–Dawley rat at 10 mg/kg, po”), the “unit” might contain only the compound mass (e.g., “10 mg” as in CHEMBL959131– “C_max_ in healthy male human at 10 mg”), the “unit” might be written with different capitalization (e.g., “10 mg/Kg”), the “unit” might be represented with a different syntax (e.g., “10 mpk” as in CHEMBL621614—“C_max_ was determine after peroral administration at 10 mpk in dog”) etc. This heterogeneity at each level of the respective parameters increased significantly the design of each RegEx.

### Data sets for model training and external validation

**Fig. 10:**
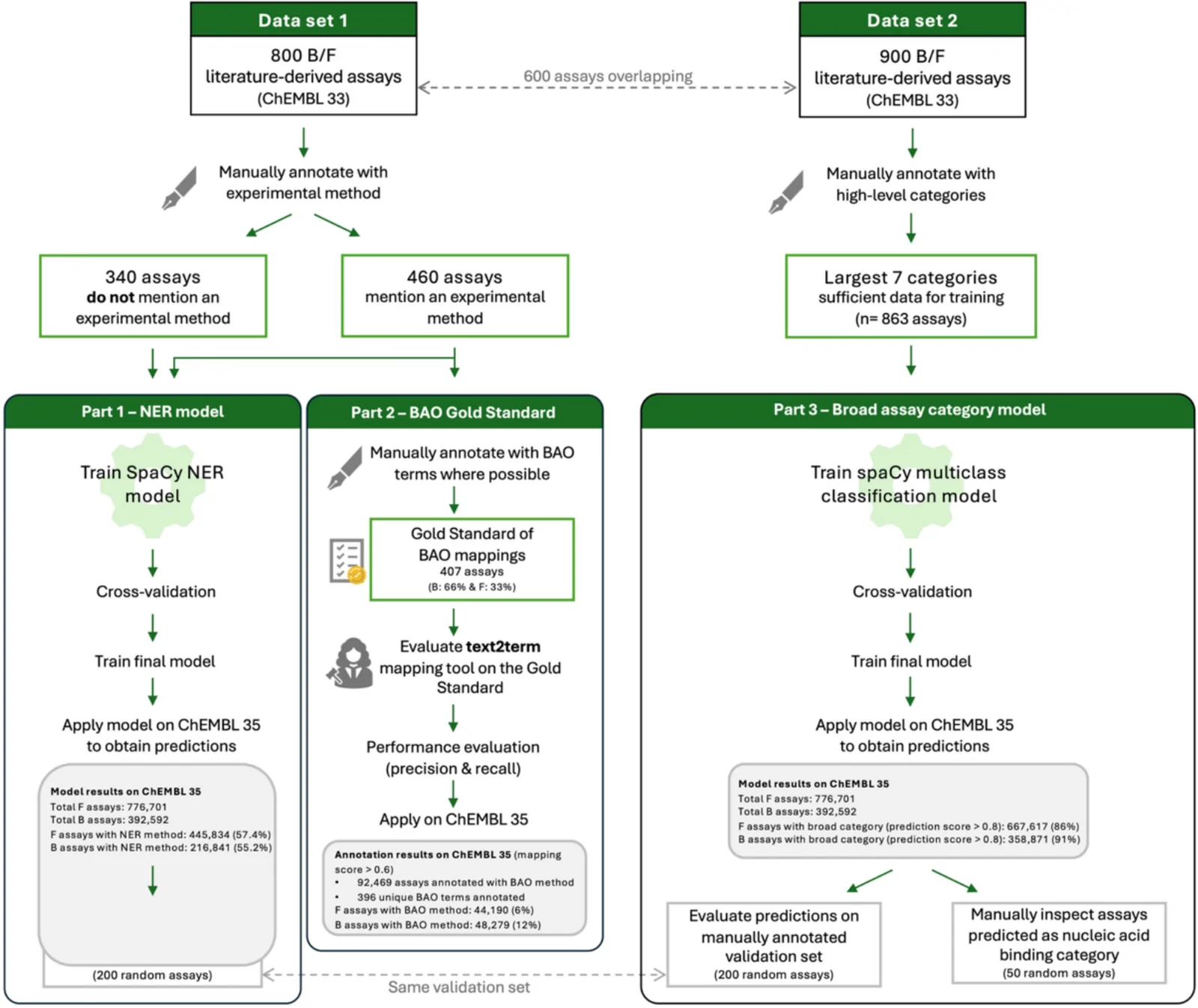
Overview of the workflow of the study and data sets used for training and evaluation

The training sets for the Natural Language Processing (NLP) models were developed through an iterative curation and annotation process, with data progressively augmented in each iteration. We initially started with a set of 300 binding assays that were chosen based on being primarily binding affinity and displacement assays. We focused on these initially as these provided a more homogeneous set of assays compared to other protein activity measurements. This provided a good starting point for developing the models as a proof of concept of the chosen methodology, before expanding the approach to more complex assays. This initial set of 300 assays is included in the training data of both the NER and broad assay category models, and it is also part of the gold standard of Bioassay Ontology (BAO) annotations [5]. The training sets were further expanded through the random sampling of binding and functional assays from ChEMBL, making sure that a variety of assays was included. The training sets were created from ChEMBL version 33 and we only included assays derived from scientific literature (src_id 1) and with a defined target (target id ! = 22226). The final manually annotated benchmark data sets have been made available for each model including assay IDs and annotations (see “Availability of data and materials” section). An overview of the datasets and general workflow used is shown in Fig. 10.

The manually annotated training data sets are comprised of the following subsets of assays:

1. Data set 1: Training data set for the NER model and gold standard data set for BAO linking (800 assays in total): 300 selected binding affinity-focused B-type assays + 300 randomly selected F-type assays + 200 randomly selected human B-type assays with target type SINGLE PROTEIN
2. Data set 2: Training data set for the broad assay aim category model (900 assays in total): 300 selected binding affinity-focused B-type assays (same as in Data set1) + 300 randomly selected F-type assays (same as in Data set 1) + 150 randomly selected B-type assays + 150 selected nucleic acid B-type assays. Thus, 600 assays are overlapping with Data set 1. Only 863 of these 900 assays were finally used for training the broad assay aim category model (comprising the seven largest categories).

The training sets for the NER and broad assay category models were annotated by two independent biological curators and the agreed consensus annotations were used for training. For the NER model, this involved identifying the specific tool, instrument, assay methodology, or kit used to measure the experimental read-out and labelling this as the "experimental method" entity respectively. A general structure for the experimental set-up described in the assay was observed. A method indicator term typically followed the annotated method, e.g., “method”, “assay”, “analysis”, “test”, “-graphy” or “-metry”, unless an acronym was annotated, such as “ELISA” (Enzyme-Linked Immunosorbent Assay), where this term then becomes part of the acronym. Detailed annotation rules for the NER gold standard data sets can be found in Supplementary Method S1. 340 of the assays in the NER training set do not mention an experimental method, but were still included in the training data set for the NER model so that the model learns to identify the lack of an experimental method.

The gold standard data set for BAO linking was annotated with terms from BAO ontology version 2.8.12 by one biological curator. Of the 460 assays in the gold standard for which an experimental method has been identified successfully, only 53 could not be mapped to a corresponding term in BAO, leaving 407 to form the gold standard. This set of 407 assays consists of ~ 66% B-type assays (n = 270) and ~ 34% F-type assays (n = 137).

The performance of the final NER and broad assay category models was also evaluated on an independent validation set of 200 randomly selected assays from ChEMBL 35. This dataset was created using stratified sampling to include representative fractions of B- and F-type assays from source_id 1 (“Scientific Literature”), excluding any assays used for training and excluding assays without an assay description or an undefined target. This resulted in 132 F-type and 68 B-type assays. The assays were manually annotated with the experimental method and broad assay category in a blinded fashion (i.e., without access to model predictions), and then systematically compared to the model outputs.

Due to the low number of assays for the nucleic acid binding category in the independent validation set of 200 assays, we further inspected the performance of the model for this category by randomly selecting 50 assays from ChEMBL 35 that were predicted as being nucleic acid binding, while excluding any assays used for training the model, and reviewing the predictions manually.

### NER model for experimental method identification

To automatically identify experimental methods within assay descriptions, a custom Named Entity Recognition (NER) model was developed using the spaCy (version 3.7.5) natural language processing library in Python. Specifically, the development process adapted a template project from Explosion AI's projects repository (available on GitHub [37]). This template provided a robust framework for creating a custom NER model, which was then tailored to the nuances of assay description texts. The underlying language model utilized was en_core_web_md (version 3.7.1), chosen for its balance of size, speed, and performance on general English text, which forms the basis of scientific assay descriptions.

The training and evaluation pipeline was managed through a series of Jupyter notebooks. Initially, the annotated dataset (assays_data.csv) was processed into spaCy's required.jsonl format, creating distinct training (assays_train.jsonl) and testing (assays_eval.jsonl) sets. To ensure robust performance assessment and mitigate potential biases from a single data split, a repeated k-fold cross-validation strategy was employed using the ner_assays_cv.ipynb notebook. This involved an 80%/20% random split of the annotated data, with the process repeated five times, resulting in a total of 25 folds. Performance metrics, including precision, recall, and F1-score for the "method" entity, were calculated across these folds (results stored in cv_metrics.csv).

Following cross-validation, the final NER model destined for application, was trained using the entire annotated dataset of 800 assays. This was accomplished using the ner_assays_model.ipynb notebook, ensuring the model learned from all available curated examples. The resulting trained model (best-performing iteration saved as model-best) was then utilized for inference on new, unannotated assay descriptions via the ner_assays_runner.ipynb notebook. The development environment relied on Python 3.12.3, with specific package dependencies managed through a virtual environment and detailed in the requirements.txt file. For more details check the project's associated code (see Data availability section).

External performance was tested on the independent stratified, random test set of 200 samples by labeling the samples as true positive (TP) samples, false negative (FN) samples, true negative (TN) samples, false positive (FP) samples, and mismatches. These are defined as follows:

- TP samples: the assay description has a method, and the model correctly identified it.
- FN samples: the description has a method, but the model failed to identify it.
- TN samples: the description does not contain a method, and the model correctly did not identify one.
- FP samples: the description does not contain a method, but the model incorrectly identified one.
- Mismatches: cases where there was a method detected but it is a mismatch between the model prediction and the curated annotation, such as missing or extra tokens.

The precision of the results was calculated as TP / (TP + FP) and the recall was calculated as TP / (TP + FN). The F1-score was calculated as the harmonic mean of precision and recall: 2 × (precision × recall) / (precision + recall).

### Broad assay aim classification model

Initially the creation of the training set for the broad assay category started with 300 affinity-focused binding assays—a less diverse subset of the B-type assay category for initial exploration. These assays were manually inspected and grouped into higher-level categories with the aim of providing more granular but still high-level categories compared to the current B-type and F-type categories. During this first-pass annotation, it became clear that the “nucleic acid binding” category was relatively small (n = 26 assays) but these numbers appeared borderline. To rescue this category for inclusion in model development we decided to manually select an additional 150 nucleic acid binding assays to balance the training data for this category in the training set. Next, these assays were further complemented by 150 randomly selected binding assays to reflect the full diversity of B-type assays, and 300 randomly selected functional assays to give a total of 900 annotated assays. These 900 assays that formed the final training set were inspected as a whole and manually grouped into higher-level categories. The manual grouping includes 15 different categories (see “Results” section). Seven of these categories included fewer than ten assays which is insufficient for modelling. As such, these were excluded along with the “unclear” category. Detailed annotation rules for the broad assay aim category gold standard data set can be found in Supplementary Method S2. This left seven categories with enough assays for modelling. The total number of assays included in the training set was 863 across these seven categories. A spaCy multiclass text classification was trained using the TextCatEnsemble.v2 architecture, which is an ensemble model of a linear bag-of-words model and a Tok2Vec neural network model. Repeated cross-validation was used to estimate the model performance using the scikit-learn RepeatedKFold function which results in different randomisation for each repeat. Using an 80–20% random split (n_splits = 5), repeated five times (n_repeats = 5), resulted in a total of 25 folds. The final model was trained using 100% of the training data and applied on ChEMBL 35. To evaluate the predictions on the validation set of 200 independent randomly selected assays, the predicted category for each assay was taken to be the assay category with the highest prediction score.

### Evaluation of the text2term ontology mapping tool

The “text2term” mapping tool [35] is a Python package for mapping biomedical descriptions to ontology terms. It provides annotations based on the Term Frequency-Inverse Document Frequency (TF-IDF) method that scores the importance of a token in a document relative to a background corpus of documents. In the text2term implementation, the ontology term labels and synonyms are considered a “document”, and the tool then computes a vector similarity between the input string and the ontology concept. Text2term was used programmatically to submit the method terms for each assay in the BAO gold standard for annotation using a local OWL file of BAO version 2.8.12 downloaded from BioPortal [38]. The mapping on the gold standard was performed using the following settings: min_score = 0.02, max_mappings = 50 and term_type “OntologyTermType.CLASS”. The performance was programmatically evaluated based on the BAO IDs in the manually annotated gold standard data set compared to the suggestions from the text2term tool. Performance was calculated as follows:

- TP samples: the BAO ID suggested by the text2term tool matches the term in the gold standard data set.
- FP samples: the BAO ID suggested by the text2term tool is not present in the gold standard data set.
- FN samples: the BAO ID is present in the gold standard data set but not suggested by the text2term tool.

Precision, recall, and F1-score were calculated as outlined above. Precision and recall were subsequently calculated at different mapping score thresholds returned by the text2term tool, and used to plot precision-recall curves.

### Text editing and review

Portions of the manuscript text were refined for clarity using ChatGPT (OpenAI, GPT-5). The authors verified all AI-generated suggestions and are responsible for the final content.

## Supplementary Information

Supplementary Material 1.

## Data Availability

Two flat files containing the annotations of the NER and the broad assay aim classification model are available from the ChEMBL FTP server: https://ftp.ebi.ac.uk/pub/databases/chembl/assayannotation/ The curated gold standard data sets as well as code for the NER model and the broad assay aim model is available here: https://github.com/chembl/chembl_assay_annotation Code for the PK/PD parameter extraction is available here: https://github.com/chembl/chembl-pk-parameter-regex.
