# Supplementary material for: Integrating artificial intelligence and manual curation to enhance bioassay annotations in ChEMBL
Source: J Cheminform. 2026 Feb 12;18:24. doi: 10.1186/s13321-026-01165-x (PMC12903245; doi:10.1186/s13321-026-01165-x)
Supplement: Supplementary file 1 [file 13321_2026_1165_MOESM1_ESM.pdf]

# - Supplementary Material -

Enhancing bioassay annotations in ChEMBL with Artificial Intelligence

Ines Smit,[1,$] Melissa F. Adasme,[1,$] Emma Manners,[1,$] Sybilla Corbett,[1] Nicolas Bosc,[1] Hoang-My-Anh Do,[1] Andrew R Leach,[1,&] Noel O'Boyle,[1] Barbara Zdrazil[1,*]

[1]*European Molecular Biology Laboratory, European Bioinformatics Institute (EMBL-EBI), Wellcome Genome Campus, Hinxton, Cambridgeshire, CB101SD, UK*

*Correspondence to: *bzdrazil@ebi.ac.uk*

[$]*These three authors contributed equally*

[&]*Present Address: LifeArc, Accelerator Building, Open Innovation Campus, Stevenage SG1 2FX, U.K*

Supplementary Figures

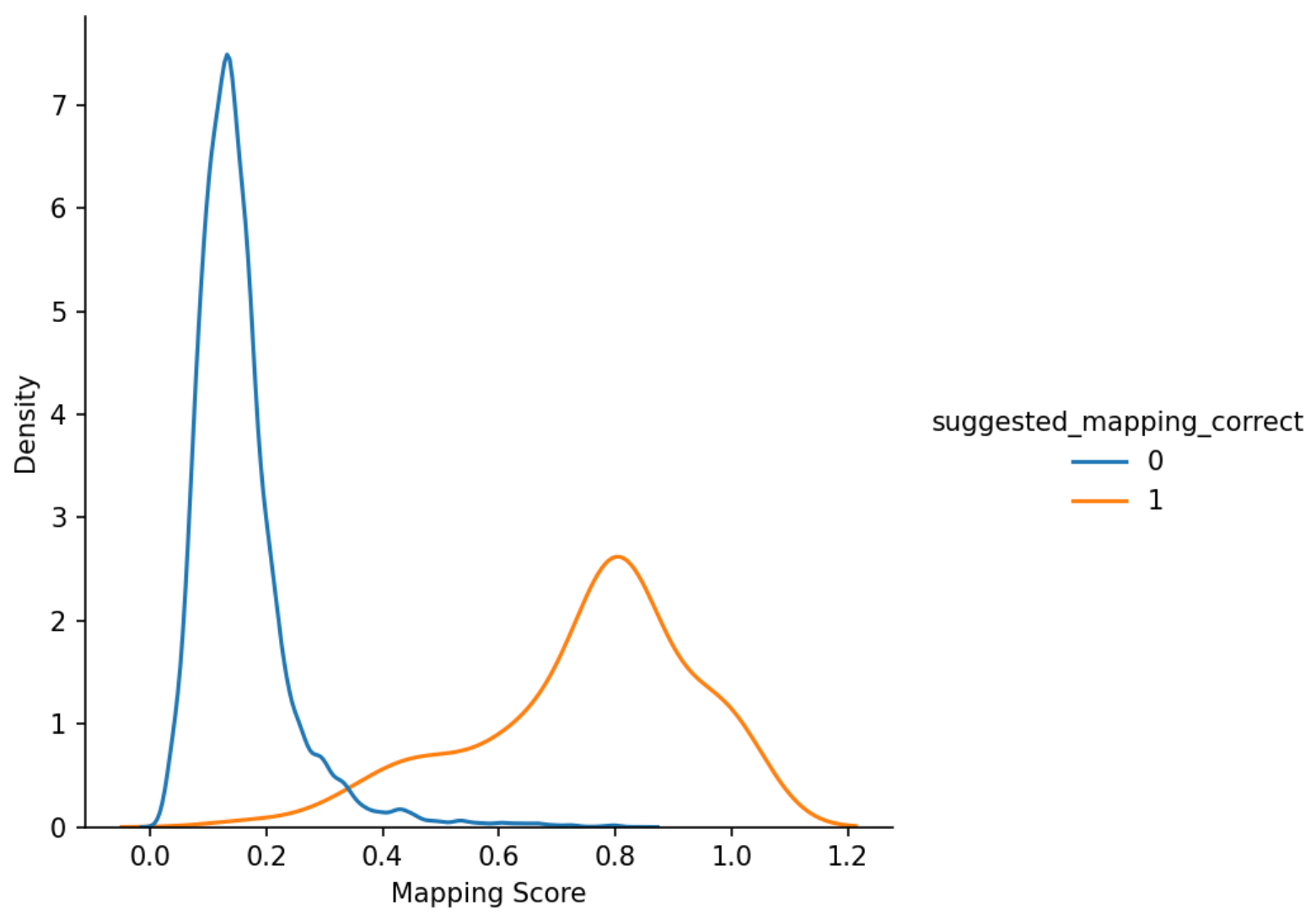

Supplementary Figure S1. Mapping Scores for ontology linking suggestions by correctness according to the Gold Standard. Mapping Scores for the ontology mapping suggestions provided by text2term for the assay ID-method pairs in the Gold Standard data set, showing good separation between suggestions that were deemed correct and incorrect based on comparison with the manually annotated Gold Standard data set.

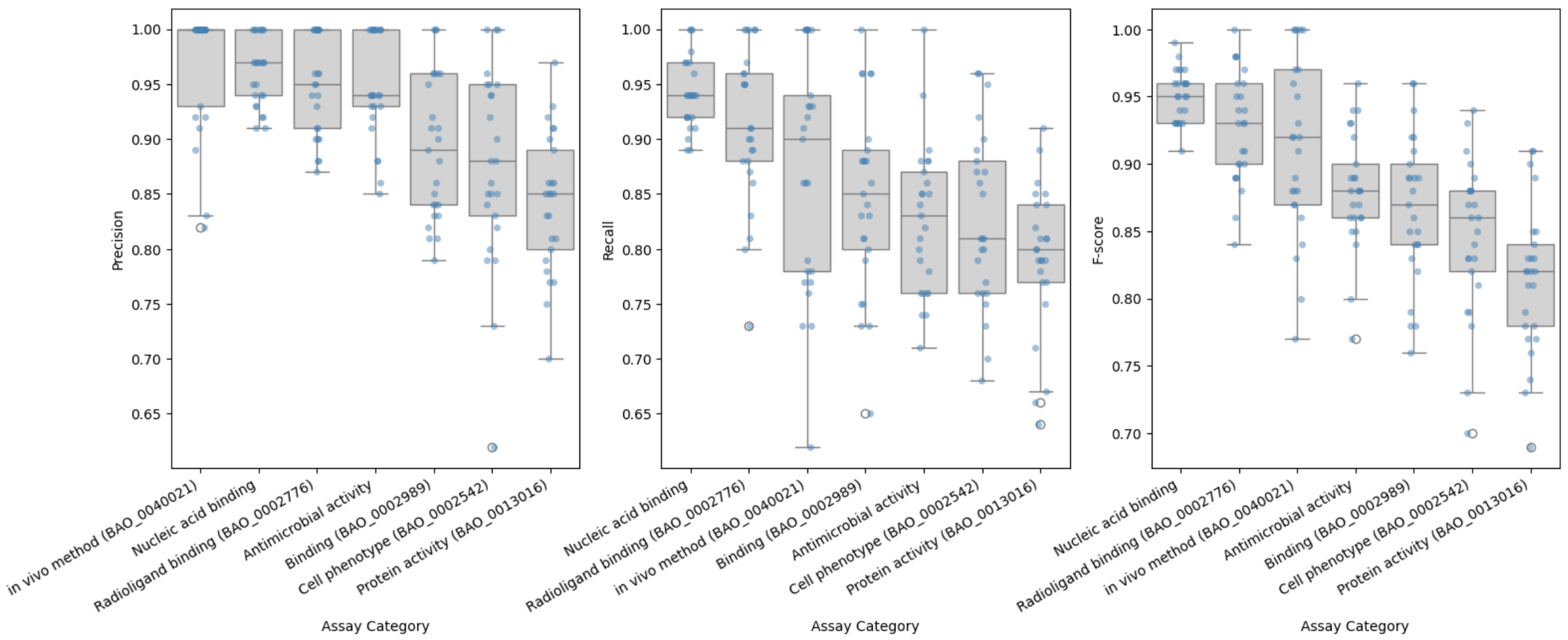

Supplementary Figure S2. Distribution of precision, recall and F1-score in cross-validation. During repeated 80-20 split cross-validation across 25 folds for the broad assay aim category model.

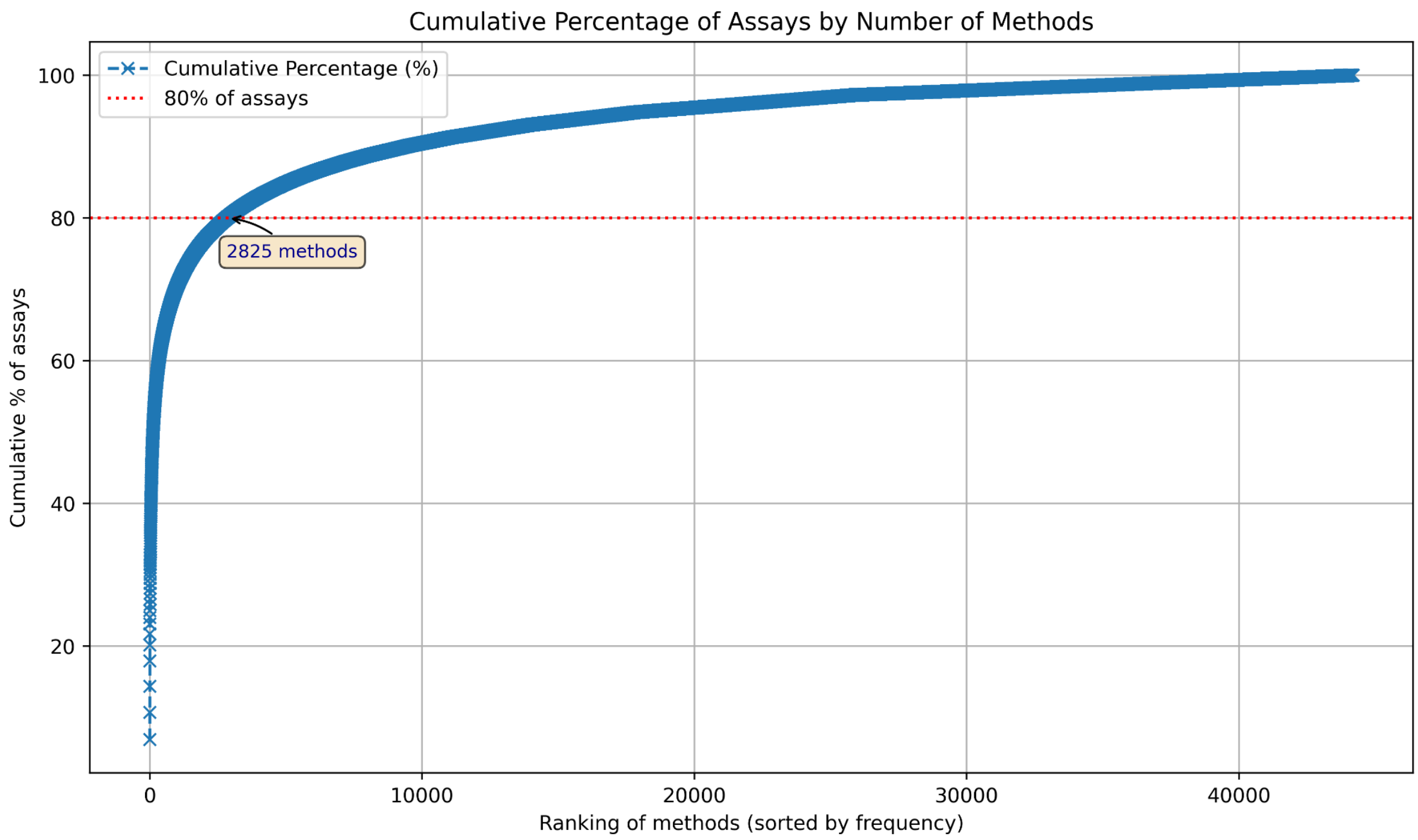

Supplementary Figure S3. Cumulative distribution of assays by the number of methods. The graph displays the cumulative percentage of assays (y-axis) accounted for by an increasing number of methods, ranked by their frequency of use (x-axis). The blue line with 'x' markers indicates this cumulative percentage. The red dotted line highlights that 80% of all assays are covered by the 2,825 most frequently used methods. This demonstrates that a significant proportion of assays report a relatively small subset of experimental methods (2,825).

Supplementary Tables

Supplementary Table S1. Descriptions of BAO terms used for the BAO-format and BAO-endpoint annotations, taken from https://www.ebi.ac.uk/ols4/ontologies/bao/

Supplementary Table S2. The precision, recall and f-score in cross-validation of the NER model.

Supplementary Table S3. 44,185 unique terms identified for the experimental method annotation by the NER model, sorted by frequency.

Supplementary Methods

Supplementary Method S1: Annotation rules for annotating the "Experimental Method".

To identify entities related to the experimental set-up within assay descriptions, a set of 800 assays was annotated with methodology-related terms.

A general structure for the experimental set-up described in the assay was observed:

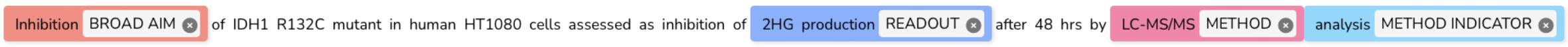

A method indicator term typically followed the annotated method e.g. method, assay, analysis, test, -graphy or -metry, unless an acronym was annotated, such as ELISA (Enzyme-Linked Immunosorbent Assay), where this term then became part of the acronym.

The goal for the NER model was to annotate the tool, instrument, assay method or kit employed to measure the experimental read-out.

Annotation of terms was performed according to the following general rules for diverse assays:

- The method is defined as the tool used to measure the assay readout.
- The method was almost always followed by method "indicator" terms and these were also annotated; these were typically "method", "assay", "analysis", "test" (assay|method|analysis|scopy|metry|test|count(ing|er)|phoresis|technique|graphy|profiling|experiment), but these were occasionally embedded into an acronym. Only occasionally were assay methods provided without one of the above terms.

  For example:

  Inhibition of IDH1 R132C mutant in human HT1080 cells assessed as inhibition of 2HG production after 48 hrs by LC-MS/MS analysis

  LC-MS/MS analysis was annotated rather than LC-MS/MS.

- The method entity terms must be adjacent and must also be an exact match to the assay description. Implied or split methodology terms were not annotated.

  For example:

  Agonist activity at human RXRalpha transfected in human HEK293T cells co-expressing pFR/pRL-Luc at 30 uM incubated for 14 to 16 hrs by hybrid reporter gene assay relative to bexarotene

Annotated "hybrid reporter gene assay" but additional terms related to the specific luciferase reporter were not captured as part of this method. In theory, the substrate would be captured if the terms were adjacent, e.g., “luceriferase reporter gene assay”.

- If no details were provided then the extracted entity field was left as null.
- The readout or type of measurement/endpoint (i.e. IC50, CC50) was not annotated and is instead recorded separately in the ChEMBL ACTIVITIES table as part of the bioactivity data.

For example:

Binding affinity to human recombinant 5-HT2A assessed as inhibition constant

Inhibition constant was not annotated.

Potentiation of erythromycin-induced antibacterial activity against Enterococcus faecium ATCC 700221 assessed as minimum modulatory concentration up to 32 uM measured after 16 hrs in presence of MIC/4 erythromycin

Minimum modulatory concentration was not annotated.

- Terms relating to the overall experimental aim were not annotated since the "broad" method will annotate these separately. For the NER model, only specific method terms that provide additional information over the broad categories were annotated during curation.

For example:

Binding affinity determined by measuring its ability to displace [3H]N-0437 radioligand in CHO-K1 cells on Cloned Human Dopamine receptor D2

Not annotated with an entity. This is a radioligand displacement assay with no additional, specific method details reported that measure the readout; the broader experimental aim (radioligand displacement assay) is captured as part of the broad category model. The assay also lacks a method "indicator" term.

Antiprotozoal activity (functional assay) using Mauden-Downing bovine kidney (MDBK) host cells infected with Eimeria tenella

Displacement of [3H]estradiol from human recombinant estrogen receptor alpha by radiometric assay relative to estradiol.

These assays were not annotated with an entity. The terms "functional assay" and "radiometric assay" include the method indicator term "assay" but these terms do not provide details on the technique used to measure assay readout and provides no additional information beyond that already captured as part of the broad model.

Competitive inhibition of human mTOR using 4EBP1 as substrate in presence of [33gammaP]-ATP after 120 mins by filter binding method.

Annotated with "filter binding method". In this assay, although "binding method" is not informative, the term "filter" provides some additional insight into the assay set-up and was annotated.

- Substrates/other chemical entities were not annotated. Whilst these could be useful in determining the assay set-up (e.g. radioligand, enzyme substrate/cofactor etc.) these are not tools to measure the assay readout and are often split across assays. The exception was where the substrate was part of an established technique.

For example:

Agonist activity at human 5HT1A receptor expressed in CHO cells by [35S]GTPgammaS binding assay

Annotated [35S]GTPgammaS binding assay. Whilst "binding assay" is not informative, the substrate used provides additional detail on the assay set-up.

Positive allosteric modulator activity at human MRGPRX1 transfected in HEK293 cells in presence of BAM8-22 by Fulo4AM dye based FLIPR assay

Annotated the Fulo4AM dye since these terms were adjacent to the Fulo4AM dye based FLIPR assay method and provided additional information on the assay set-up.

Inhibition of CMFDA binding to human N-terminal 6x-His-tagged GSTO1-1 at 10 uM preincubated for 30 mins followed by CMFDA addition and measured after 30 mins in presence of 10 uM MnCl2 by in-gel fluorescence binding assay

Not annotated. CMFDA substrate not annotated since it's separate from the method term, however it is relevant to the in-gel fluorescence assay.

Binding constant for DNA by ethidium bromide displacement

The ability to reversibly bind to DNA using ethidium bromide displacement assay was reported as apparanet DNA binding constant for Coliphage T4 DNA

Binding constant for DNA by ethidium bromide displacement

Poison activity at recombinant human topoisomerase-2alpha expressed in Saccharomyces cerevisiae JEL1 harboring topoisomerase1 deletion mutant assessed as increase in double stranded DNA cleavage at 1 mM using supercoiled pBR322 DNA as substrate preincubated for 1 to 5 mins followed by substrate addition measured after 6 mins by ethidium bromide staining based agarose gel electrophoresis

Annotation was only performed when the compound "ethidium bromide" was coupled with a specific method (" ethidium bromide staining based agarose gel electrophoresis" but not "displacement") which provided information about the tool used.

- Supplier platforms were not annotated as assay methods.

For example:

Inhibition of human CREBBP (A1040 to G1161 residues) at 10 uM by DiscoverX assay relative to control.

The supplier DiscoverX was not annotated.

Supplementary Method S2: Curation rules for annotating the "Broad assay aim".

To broadly assign assays to categories, assay descriptions were reviewed and annotated with one of 15 labels that reflected the most common assay set-up for binding and functional assays.

- Protein activity
- Binding affinity, displacement, competition
- Radioligand competition, displacement, binding
- Cell phenotype
- Antimicrobial activity
- In vivo method
- Nucleic acid binding
- Whole-organism assay
- Antioxidant activity
- Protein-protein interaction assay
- Stimulation of binding
- Binding/activity - mixed measurements
- Tissue based assay
- Prodrug metabolism
- Unclear

Categories were then mapped to the closest label from the Bioassay Ontology.

The mappings to BAO labels can be found in the following table:

**Assay Category**	**BAO Preferred Term**	**BAO ID**
Whole-organism assay	NaN	NaN
Tissue based assay	NaN	NaN
Antioxidant activity	NaN	NaN
Cell phenotype	cell phenotype	BAO_0002542
Radioligand competition, displacement, binding	radioligand binding assay	BAO_0002776
Binding affinity, displacement, competition	binding assay	BAO_0002989
Protein-protein interaction assay	NaN	NaN
Protein activity	functional target-based	BAO_0013016

In vivo method	in vivo assay method	BAO_0040021
Nucleic acid binding	NaN	No suitable ID at present
Antimicrobial activity	NaN	No suitable ID at present
Binding/activity - mixed measurements	NaN	NaN
Stimulation of binding	NaN	NaN
Prodrug metabolism	NaN	NaN
Unclear	NaN	NaN

Assays with conflicting terms or where the set-up was ambiguous were assigned as unclear. For example, "Inhibition of human recombinant NTS1 receptor at 10 uM by radioligand binding assay" contains conflicting terms referring to enzyme activity (inhibition) and also to binding methods (radioligand binding assay). Other examples include the assay " Inhibition of RTK/PI3K/AKT/mTOR signaling in human U87 cells assessed as down regulation of TRAF4 gene expression at cytotoxic IC50 measured after 48 hrs relative to control" where it was unclear if this was a functional target-based or a cell phenotypic assay.

Overall, categories such as the "cell phenotype" group include cytotoxicity, cell metabolism and other assays measuring cellular changes to protein expression. The "functional target-based" category includes enzyme activity, receptor signalling and other assays where a change in function, as a result of compound-target interactions, is measured. Notably, the annotation is somewhat dependent on the structure of the assay description and overlap is possible. For instance, target-based functional assays performed in cells could have a phenotypic readout and be classified as either "target-based functional" or "cell phenotypic" depending on the structure of the assay description. Imprecise wording or atypical assay set-up may also effect categorisation; the assay "Minimum protective concentration against beta-lactamase in Staphylococcus aureus (95 strain)" uses terms often found within in vivo assays (minimum protective concentration) but is most likely a functional target-based assay. Similar categories such as the whole-organism assay and the in vivo assay category were used to differentiate in vivo assays with model organism to other whole-organism assays with plants or microorganisms (that didn't belong to the antimicrobial category and were not classed as cell phenotype changes).

In addition to potentially overlapping assays, other challenges included differentiating assays where the information in assay descriptions was insufficient to determine if assays were simply binding or functional. The binding and target-based functional assays categories were the most difficult to

differentiate. Some compromises were made such as antagonist assays being assigned to the "protein activity" group since the direction of the effect is recorded although the assay could be binding or functional.
